# Members of the vertebrate contactin and amyloid precursor protein families interact through a conserved interface

**DOI:** 10.1016/j.jbc.2021.101541

**Published:** 2021-12-25

**Authors:** Sebastian J. Karuppan, Alex Vogt, Zachary Fischer, Aliona Ladutska, Jonathan Swiastyn, Hillary F. McGraw, Samuel Bouyain

**Affiliations:** 1 Department of Cell and Molecular Biology and Biochemistry, School of Biological and Chemical Sciences, University of Missouri-Kansas City, Kansas City, Missouri, USA; 2 Department of Genetics, Developmental and Evolutionary Biology, School of Biological and Chemical Sciences, University of Missouri-Kansas City, Kansas City, Missouri, USA

**Keywords:** amyloid precursor protein, axon, cell adhesion, cell surface protein, contactin, protein–protein interaction, X-ray crystallography, zebrafish, APLP1, amyloid beta precursor like protein 1, APLP2, amyloid beta precursor like protein 2, APP, amyloid precursor protein, BLI, biolayer interferometry, cDNA, complementary DNA, CNTN, contactin, CNTNAP4, CNTN-associated protein–like 4, FN, fibronectin type III repeat, FN1, first FN repeat, FN2, second FN repeat, FN3, third FN repeat, GABBRI, gamma-aminobutyric acid type B receptor subunit 1, GPI, glycosylphosphatidylinositol, hpf, hours postfertilization, Ig, immunoglobulin, PDB, Protein Data Bank, PTPRG, protein tyrosine phosphatase receptor type G

## Abstract

Contactins (CNTNs) are neural cell adhesion molecules that encode axon-target specificity during the patterning of the vertebrate visual and olfactory systems. Because CNTNs are tethered to the plasma membrane by a glycosylphosphatidylinositol anchor, they lack an intracellular region to communicate across the membrane. Instead, they form coreceptor complexes with distinct transmembrane proteins to transmit signals inside the cell. In particular, a complex of CNTN4 and amyloid precursor protein (APP) is known to guide the assembly of specific circuits in the visual system. Here, using *in situ* hybridization in zebrafish embryos, we show that CNTN4, CNTN5, and the APP homologs, amyloid beta precursor like protein 1 and amyloid beta precursor like protein 2, are expressed in olfactory pits, suggesting that these receptors may also function together in the organization of olfactory tissues. Furthermore, we use biochemical and structural approaches to characterize interactions between members of these two receptor families. In particular, APP and amyloid beta precursor like protein 1 interact with CNTN3–5, whereas amyloid beta precursor like protein 2 only binds to CNTN4 and CNTN5. Finally, structural analyses of five CNTN–amyloid pairs indicate that these proteins interact through a conserved interface involving the second fibronectin type III repeat of CNTNs and the copper-binding domain of amyloid proteins. Overall, this work sets the stage for analyzing CNTN–amyloid-mediated connectivity in vertebrate sensory circuits.

Trillions of synaptic connections are established with exquisite specificity during the patterning of the vertebrate nervous system. During neural development, axons follow guidance cues to extend away from their point of origin to connect to the appropriate lamina (1, 2). Once in proximity of the target area, axons select one particular cell or group of cells among many possible choices to initiate synapse formation. The specificity of axon targeting is thought to be determined, at least in part, by interactions between cell surface receptors expressed on growth cones and on target cellular surfaces. Although a mechanistic understanding of how receptors or combinations of receptors can bias connectivity toward certain targets remains incomplete, several families of cell adhesion molecules have been implicated in these processes. These include plexins and associated semaphorins, cadherins, and several immunoglobulin (Ig) superfamily proteins, such as sidekicks, as well as members of the L1 and contactin (CNTN) families (2).

Several lines of evidence have implicated CNTNs in establishing specific connectivity in sensory systems. In the mouse olfactory system, axons from olfactory sensory neurons expressing identical odorant receptors converge onto glomeruli in the olfactory bulb. In animals lacking *Cntn4*, a subset of olfactory sensory neurons innervated ectopic glomeruli in the olfactory bulb, suggesting that CNTN4 is involved in the assembly of specific olfactory circuits (3). Similarly, CNTN4 biases the arborization of a subpopulation of retinal ganglion cell axons to a region of the accessory optic system required for horizontal image stabilization (4). Aberrant dendritic arborization is also observed in subset of neurons found in the inner plexiform layer of mouse retinas lacking CNTN5 (5). Finally, in chicken embryonic retinas, CNTN1–5 are expressed in distinct sublaminae of the inner plexiform layer in a nonoverlapping pattern, and CNTN1–3 mediate specific connections between sublaminae (6). Taken together, these findings suggest that CNTNs help establish a targeting code to form specific synaptic connections in the visual and olfactory systems.

CNTNs are somewhat unique among the receptors linked to axon-target specificity because they are anchored to the cell membrane by a glycosylphosphatidylinositol (GPI) anchor. Consequently, they need to partner with a coreceptor in order to transduce signals inside the cell. Consistent with this idea, CNTN5 binds to CNTN-associated protein-like 4 (CNTNAP4) on the surface of dendrites of a retinal ganglion cell subpopulation that targets specific neurons in the inner plexiform layer (5). In addition, this receptor complex interacts homophilically with a CNTN5–CNTNAP4 complex found on the target neurons (5). In other words, CNTNAP4 functions not only as a link between CNTN5 and intracellular signaling machineries but also promotes adhesion between the ganglion cell layers and the neurons of the inner plexiform layer. In line with these findings, the targeting of retinal ganglion cell axons to the accessory optic system mediated by CNTN4 also depends on the presence of amyloid precursor protein (APP) in the same subset of axons (4). The targeting and arborization defects observed in *Cntn4*^*−/−*^ mice are essentially identical in mice lacking *App*, which is expressed ubiquitously in retinal ganglion cell axons (4, 7). Furthermore, the phenotype of *App*^*−/−*^ *Cntn4*^*−/−*^ mice is similar to *App*^*−/−*^ or *Cntn4*^*−/−*^ animals suggesting that CNTN4 and APP function in the same pathway. Because APP and its homologs, amyloid beta precursor like protein 1 (APLP1) and amyloid beta precursor like protein 2 (APLP2), have broadly overlapping functions (8) and because both APP and APLP1 interact with CNTN3–5 (9, 10), we hypothesized that coreceptor complexes formed by APPs and CNTNs could participate in the array of adhesive interactions that ensure appropriate targeting of axons and dendrites in neural sensory circuits.

As a first step toward identifying the roles that CNTN–amyloid pairs play in axon-target specificity, we decided to examine the spatiotemporal expression of genes encoding CNTN and amyloid family members as well as determine the structural basis of CNTN–amyloid interactions. Here, using *in situ* hybridizations in zebrafish embryos, we present evidence that genes encoding APLP1, APLP2, CNTN4, and CNTN5 are broadly expressed in olfactory areas and characterize previously unreported interactions between CNTN4, CNTN5, and APLP2. Using biochemical and structural approaches, we also show that the E1 domains of APP, APLP1, and APLP2 interact with the second fibronectin type III repeat (FN) repeat found in CNTN3–5 through a conserved interface. Overall, these studies raise the possibility that interactions between CNTN and amyloid family members may participate in the patterning of olfactory sensory tissues in vertebrates and provide tools to dissect these processes at a molecular level.

## Results

### Expression of genes encoding CNTN and amyloid family members in zebrafish embryos

**Figure 1:**
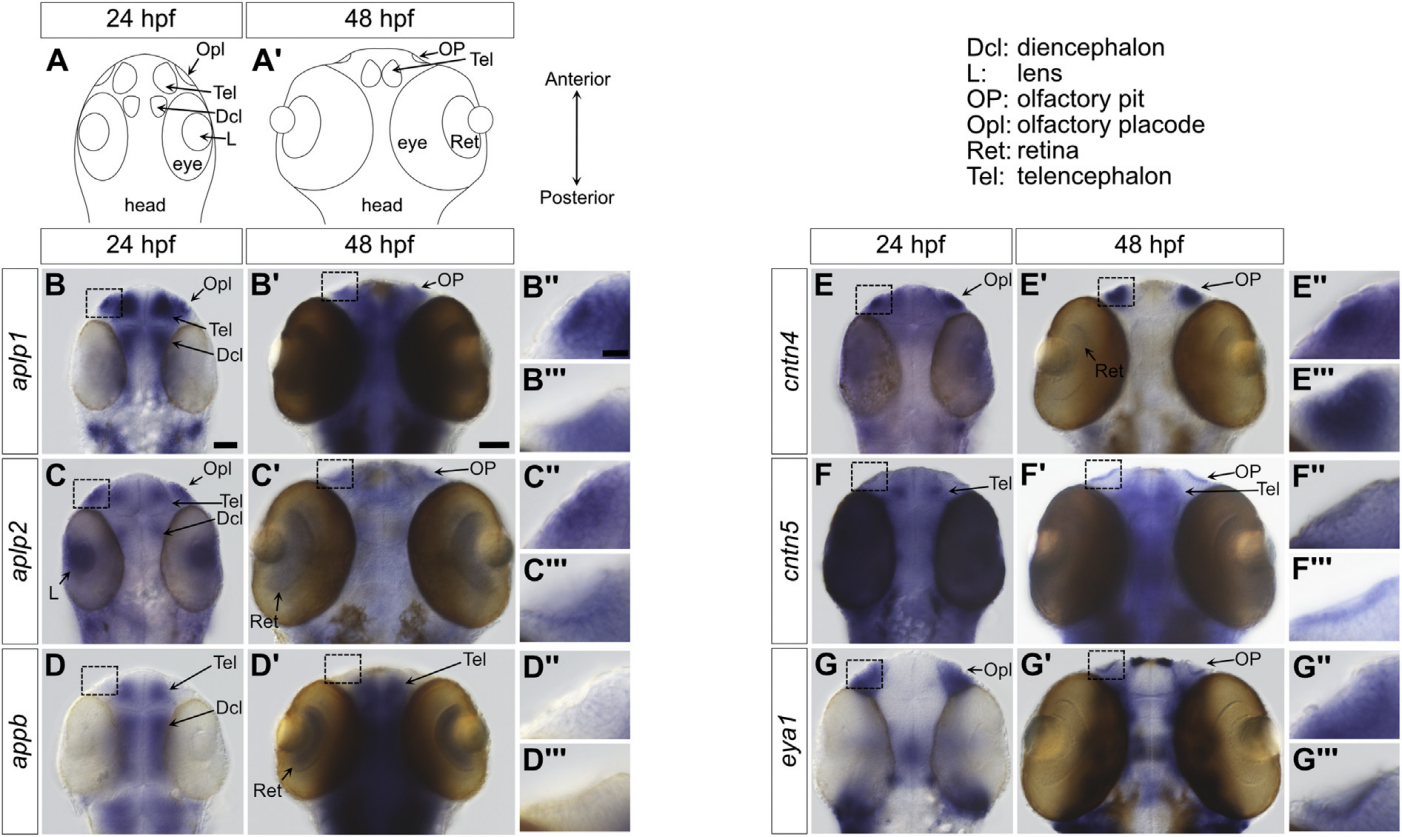
**Expression patterns of genes encoding amyloid and CNTN family members in zebrafish embryos.***A* and *A′*, cartoons representing the dorsal views of heads from zebrafish embryo at 24 and 48 h post fertilization (hpf). These views are similar to the whole-mount *in situ* hybridization analyses of amyloid and CNTN family members shown in *B*–*G* and *B*′–*G*′. *B*–*B'''*, at 24 hpf, the *colorimetric blue signal* indicates the presence of *aplp1* mRNA in the olfactory placode, telencephalon, and diencephalon. Although more widespread, the colorimetric signal remains in the olfactory placode at 48 hpf. *C*–*C'''*, at 24 hpf, *aplpl2* is expressed in the olfactory placode, eye lens, telencephalon, and diencephalon. The expression remains localized to these general areas at 48 hpf. *D*–*D'''*, at 24 hpf, *appb* is expressed mostly in areas of the central nervous system and specifically in the telencephalon and diencephalon. It can also be detected in retinas at 48 hpf. However, no signal is observed in olfactory areas. *E*–*E'''*, the expression of *cntn4* is prevalent in the olfactory placode (24 hpf) and the olfactory pit (48 hpf) although a weak signal can be observed in the retina at 48 hpf. *F*–*F'''*, *cntn5* is found in the olfactory pit (48 hpf) and the telencephalon (24–48 hpf). *G*–*G'''*, the expression of the gene *eya1*, which is known to be expressed in the olfactory placode and olfactory pit, is shown for comparison (18). For *aplp1*, *B''* and *B'''* show detailed views of the olfactory regions denoted by *boxes* in *B* and *B′*, respectively. This panel arrangement is repeated for *aplpl2*, *appb*, *cntn4*, *cntn5*, and *eya1*. The scale bar represents 25 μm (24 hpf), 50 μm (48 hpf), and 10 μm in the detailed views of olfactory regions. CNTN, contactin.

We first sought to determine the spatiotemporal expression patterns of CNTNs and amyloids to help narrow down the circuits that these receptors help pattern. We thought that zebrafish was an ideal organism to carry out this work because (i) the anatomy of the zebrafish nervous system closely resembles the human one (11), (ii) pathways underlying the development of the nervous system are broadly conserved between zebrafish and humans (12, 13), (iii) ∼70% of human genes, including *app*, *aplp1*, *aplp2*, and *cntn1–5*, have clear orthologs in fish (14), and (iv) embryos are translucent and easily accessible. Zebrafish underwent a gene duplication event that resulted in two copies of the *app* gene, *appa* and *appb* (15, 16), with the APPb sequence being more closely related to the APP sequence expressed in human brains (17). Unfortunately, our initial attempts to detect APPb and CNTN4 using antibodies obtained after inoculating rabbit and chicken with purified proteins, respectively, were unsuccessful. We thus decided to characterize the spatiotemporal expression of *aplp1*, *aplp2*, *appb*, *cntn4*, and *cntn5* during the first 48 h of embryonic development using antisense RNA probes (Fig. 1). Furthermore, because CNTN4 has been implicated in the assembly of olfactory circuits, we used an antisense RNA probe against the transcriptional coactivator *eya1* that is expressed in the olfactory placode, a precursor of the olfactory epithelium (18).

At 24 h postfertilization (hpf), *aplp1* mRNA could be detected in the olfactory placode, the telencephalon (where neurons of the olfactory bulb project to), and the diencephalon (Fig. 1, *A*, *B*, and *B''*). Expression of *aplp2* overlapped with that of *aplp1*, although the presence of *aplp2* mRNA could be also detected in the lens (Fig. 1*C*). At 48 hpf, *aplp1* is more broadly expressed in the developing brain and olfactory pit (Fig. 1*A'*) and *aplp2* is localized to the retina and olfactory pits (Fig. 1*C'*). Although the expression of *aplp2* in the olfactory pits appears restricted, it seems to correspond to the site of expression of placodal marker *eya1* in that region (Fig. 1, *G'* and *G''*). This result is consistent with previous gene expression studies in 36 hpf zebrafish embryos (19) as well as the expression of APLP2 in retinas and olfactory epithelia (20, 21)—although it should be noted that these experiments were conducted in adult mice. In contrast, *appb* was not detected in the olfactory placode or olfactory pit (Fig. 1, *D''* and *D'''*), and its expression overlapped with the structures of the central nervous system, including the telencephalon and diencephalon and the retina (Fig. 1, *D* and *D'*).

In contrast to amyloid family members, the expression of *cntn4* is almost exclusively localized to the olfactory placode (24 hpf) and the olfactory pits (48 hpf) (Fig. 1, *E*–*E'''*). We detected only weak expression of *cntn4* in the retina (48 hpf), but the visual system is not functional at 48 hpf (22), and the pigmentation of the eyes might obfuscate the colorimetric signal indicating the presence of *cntn4* mRNA. Future work at later developmental time points will help address the role of *cntn4* in retinal patterning. Finally, mRNA for *cntn5* can be detected first in the telencephalon at 24 hpf (Fig. 1, *F* and *F''*). The signal then localizes to the olfactory pits in 2-day embryos and seems to match the expression of *eya1* (Fig. 1, *F'*, *F'''*, *G'*, and *G'''*). Taken together, these *in situ* hybridization patterns indicate that the expression of *cntn4* broadly overlaps with *aplp1* and *aplp2* in the olfactory placode and olfactory pits. As for *cntn5*, it overlaps with *aplp1*, *aplp2*, and *appb* in the telencephalon at 24 hpf and with *aplp1* and *aplp2* in the apical aspect of the olfactory pits at 48 hpf. The presence of CNTN4 in olfactory pits is consistent with previous work indicating that it is expressed in a subpopulation of mouse olfactory sensory neurons and guides their axons toward specific glomeruli of the olfactory bulb (3). Expression of *Cntn5* has been reported during development of the rat olfactory bulb (23), which agrees with the expression patterns we find in zebrafish embryos. In contrast, CNTN5 has not previously been reported in olfactory pits, to the best of our knowledge. Overall, these results raise the possibility that CNTN4, CNTN5, APLP1, and APLP2 might be involved in the patterning of olfactory tissues.

### Interactions between APP, APLP1, APLP2, and CNTN3–5

**Figure 2:**
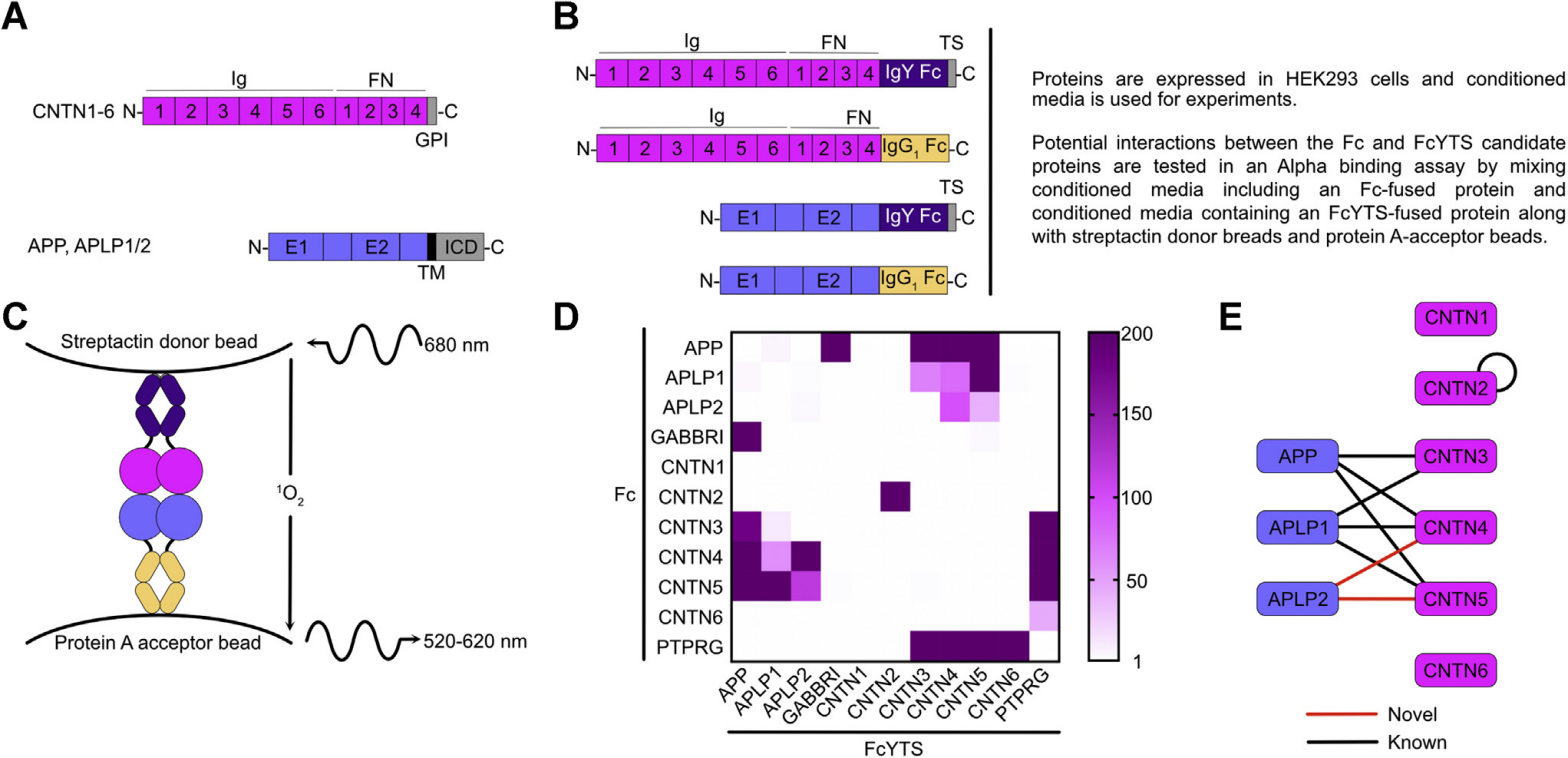
**Overview of binding assay to analyze CNTN–amyloid interactions.***A*, domain organization of CNTNs and amyloid proteins. CNTN1–6 include six immunoglobulin (Ig) domains, four FN domains, and are tethered to the cell membrane by a GPI anchor. APP and its homologs, APLP1 and APLP2, include two globular domains in their extracellular region designed E1 and E2. The E1 domain itself includes two subdomains, a growth factor–like domain followed by a copper-binding domain. The ectodomains of amyloid proteins are followed by single transmembrane helix (TM) followed by an intracellular domain (ICD). *B*, design of constructs used in the binding assay. The ectodomains of CNTNs and amyloids are fused to the Fc domain of human IgG_1_ or a protein designated FcYTS that includes (i) the Cν3 and Cν4 domains of chicken IgY and (ii) a Twin-Strep (TS) tag for detection with streptactin. *C*, in AlphaScreen assays, excitation of donor beads at 680 nm triggers the release of a singlet oxygen. An acceptor bead hit by this highly reactive molecule emits a signal between 520 and 620 nm. Because the half-life of the singlet oxygen is limited, a luminescent signal is only obtained when the donor beads and acceptor beads are within 200 nm. Here, the binding of the FcYTS fusion protein immobilized on donor beads to the Fc fusion protein bound to acceptor beads brings the two beads in proximity. *D*, results of the binding assay shown in a heat map representation. Raw signals are provided in Fig. S1. The scale indicates the value of the signal for the protein pair protein 1-FcYTS/protein 2-Fc divided by the signal measured for the protein 1-FcYTS/Fc only pair. *E*, summary of the interactions identified in the extracellular interaction screen. *Black* and *red lines* denote known and novel interactions, respectively. The positive controls, GABBRI and PTPRG, have been omitted for clarity. APLP1, amyloid beta precursor like protein 1; APLP2, amyloid beta precursor like protein 2; APP, amyloid precursor protein; CNTN, contactin; FN, fibronectin type III repeat; GABBRI, gamma-aminobutyric acid type B receptor subunit 1; GPI, glycosylphosphatidylinositol; PTPRG, protein tyrosine phosphatase receptor type G.

**Supplemental Figure S1:**
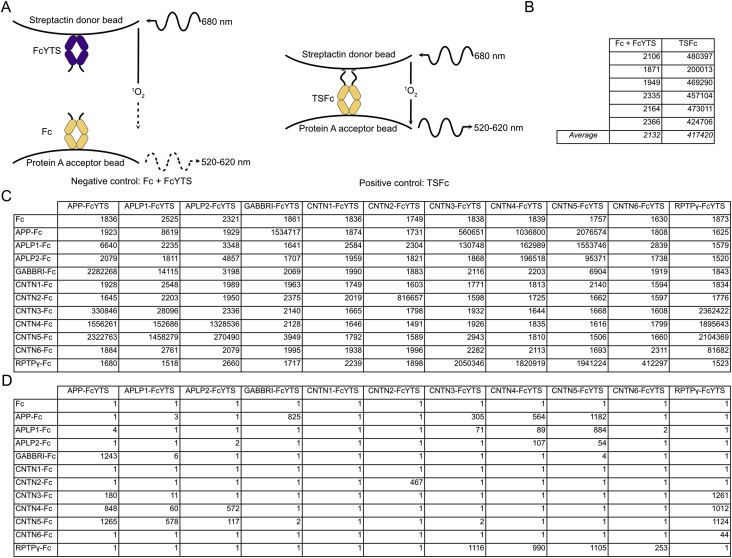
**Results of the extracellular interaction screen.***A*, conditioned media obtained after transfection of HEK293 cells with expression vectors including only the Fc and FcYTS tags serve as negative controls. Conversely, conditioned media obtained after transfection of a vector that directs the expression of a Twin-Strep tag fused to human IgG_1_ Fc – designated TSFc – serves as a positive control. *B*, luminescent signals measured for the negative and positive controls are shown in the table. These values are averaged and the ratio of these two averages (here, ∼200) was used to establish the scale for the heatmap shown in Figure 2*D*. *C*, luminescent signal measured for each tested potential receptor/ligand pair. Proteins fused to FcYTS are shown in the top row while proteins fused to Fc are shown in the leftmost column. *D*, the signals obtained for each pair are normalized against the signal obtained for human IgG_1_ Fc (Fc). For example, the signal for the APP-FcYTS/CNTN4-Fc pair (1,556,261) is divided by the signal obtained for APP-FcYTS/Fc (1836) and the result (848) is shown without any significant digit. Some of the measured signals exceed the value of 200 obtained for the positive controls. We surmise that the small size of the positive control molecule compared to bone fide receptor/ligand pairs tagged with Fc and FcYTS might bring the donor and acceptor beads too close to one another, thus leading to steric interference.

Previous reports have provided strong biochemical evidence for interactions between APP, APLP1, and CNTN3-5 (9, 10, 24). However, the results of our gene expression studies indicate that *aplp2* is expressed in olfactory areas during the first 2 days of development where it appears to overlap first with *cntn4* and then with *cntn5*. We thus decided to investigate potential associations between APLP2 and CNTN family members given the overlapping physiological roles played by APP, APLP1, and APLP2 (16). We designed an extracellular interactome assay to test for pairwise interactions between amyloids and CNTNs as well as to set the stage for future investigations of amyloid and CNTN-binding partners (Figs. 2 and S1). A common guiding principle in such assays is to express target proteins as oligomers to increase the avidity of potential interactions (25, 26, 27, 28). In our assays, the extracellular domains of the target cell surface receptors were expressed transiently in human embryonic kidney 293 cells as fusion proteins with either the Fc region of human IgG or the Fc region of the chicken IgY fused to a Twin-Strep tag, a 28 amino acid peptide designed to bind with nanomolar affinity to an engineered form of streptavidin called streptactin (29) (Fig. 2*B*). Interactions are detected using AlphaScreen technology in which a luminescent signal is emitted when a target protein fused to the FcY-Twin-Strep tag (designated FcYTS) bound to a streptactin donor bead associates with another target protein fused to IgG Fc attached to a protein A acceptor bead (protein A does not bind to the Fc region of IgY (30)) (Fig. 2*C*).

To validate our methodology, we first monitored the self-association of CNTN2, which forms homodimers and used gamma-aminobutyric acid type B receptor subunit 1 (GABBRI) and protein tyrosine phosphatase receptor type G (PTPRG) as positive controls for interactions with APP and CNTN3–6, respectively (31, 32, 33, 34, 35). We detected interactions between the pairs CNTN2-Fc/CNTN2-FcYTS, APP-Fc/GABBRI-FcYTS, APP-FcYTS/GABBRI-Fc, PTPRG-Fc/CNTN3-6-FcYTS, and PTPRG-FcYTS/CNTN3-6-Fc (Figs. 2*D* and S1, *C* and *D*). With our experimental setup validated, we then examined potential interactions between CNTN and amyloid family members. Previous interactions between APP, APLP1, and CNTN3-5 were confirmed (9, 10). We also detected association between APLP2 and CNTN4 and CNTN5, which have not previously been reported (Fig. 2, *D* and *E*). We did not observe any interaction between APLP2 and CNTN3, however.

### Biochemical characterization of CNTN–amyloid interactions

**Figure 3:**
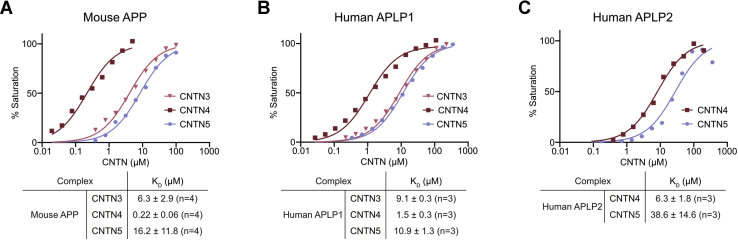
**Validation of CNTN–amyloid interactions by biolayer interferometry.** The E1 domains of APP, APLP1, and APLP2 were biotinylated in a 1:1 M ratio, immobilized onto streptavidin sensors, and titrated against varying concentrations of the FN1–FN3 region of selected CNTN proteins. One representative experiment for each series is shown in *A*–*C*, whereas a summary of the affinities measured for at least three biological replicates is shown below each curve. The plots were normalized to the maximal response. APLP1, amyloid beta precursor like protein 1; APLP2, amyloid beta precursor like protein 2; APP, amyloid precursor protein; CNTN, contactin; FN1, first FN repeat; FN3, third FN repeat.

**Supplemental Figure S2:**
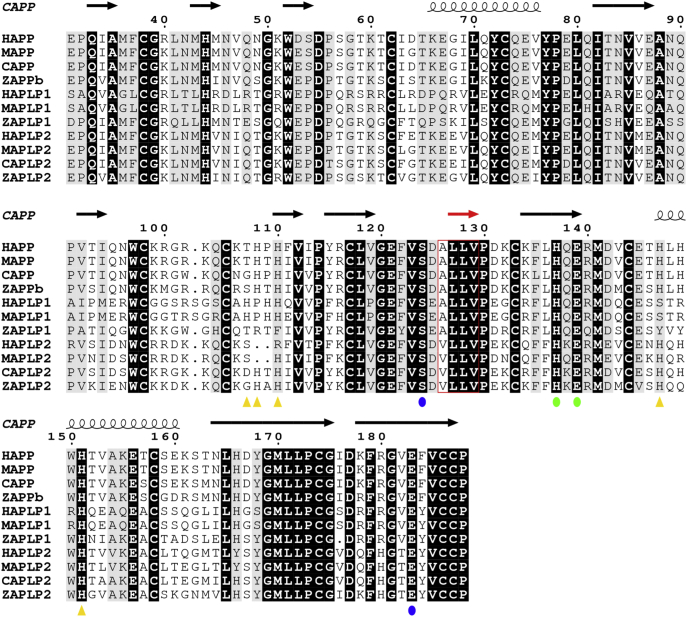
**Alignment of amino acid sequences of E1 domains for chicken, human, mouse, and zebrafish APP, APLP1, and APLP2.** The secondary structure of chicken APP is shown above the sequence and the residue numbering corresponds to chicken APP. Strictly conserved residues are shown in black, while similar positions are shaded grey. Residues found in a β-strand involved main chain/main chain hydrogen bond interactions with a β-strand in CNTNs are boxed in red. Blue and green dots indicate residues involved in side chain/main chain and side chain/side chain hydrogen bonds, respectively. Gold triangles denote copper-binding residues.

**Supplemental Figure S3:**
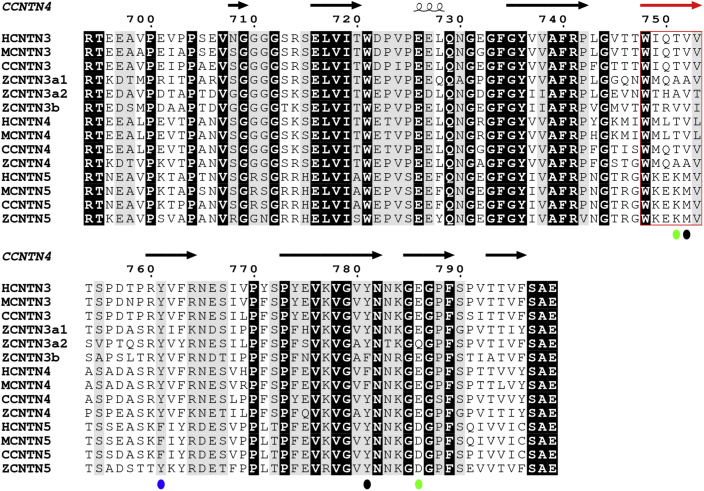
**Alignment of amino acid sequences of FN2 domains of for chicken, human, mouse, and zebrafish CNTN3–5.** The secondary structure of chicken CNTN4 is shown above the sequence and the residue numbering corresponds to chicken CNTN4. Identical residues are shown in black, while similar positions are shaded grey. Blue and green dots indicate residues involved in side chain/main chain and side chain/side chain hydrogen bonds, respectively. The black dots denote residues involved in packing interactions against H137 in APP.

We decided to carry out protein-binding assays using biolayer interferometry (BLI) to confirm the results of our extracellular binding assays and characterize the interactions between CNTN and amyloid family members. Previous work has indicated that interactions occur between the copper-binding region located in the E1 domain of APP and the second FN repeat (FN2) of CNTNs (9, 10) (Fig. 2*A*). We thus set out to quantify the binding between the biotinylated E1 domain of human APP immobilized on a streptavidin biosensor tip and the first FN repeat (FN1)–third FN repeat (FN3) regions of human CNTN3–5. Unfortunately, human APP(E1) had a tendency to aggregate, so we opted to characterize interactions between the mouse APP and mouse CNTN3–5 instead (Fig. 3). The E1 domains of human and mouse APP share 97% amino acid sequence identity, whereas the FN2 repeats of mouse CNTN3, CNTN4, and CNTN5 share 93 to 99% sequence identity with their human counterparts, suggesting that findings obtained with mouse CNTN and amyloid family members could apply to the human ones (Figs. S2 and S3). The dissociation rate constants (*k*_off_) typically approached the accuracy limits of our instrument (Table S1), so the binding affinities (*K*_*D*_) were calculated by plotting the maximal signal measured at equilibrium for a series of CNTN concentrations. In this setting, CNTN4 interacts most strongly with APP (*K*_*D*_ = 0.22 μM), whereas the equilibrium dissociation constants between CNTN3 and CNTN5 are at least 30-fold higher (*K*_*D*_ = 6.3 and 16.2 μM, respectively; Fig. 3*A*). These differences in binding affinities indicate that CNTN4 may be the preferred binding partner for APP among CNTNs.

**Supplemental Figure S4:**
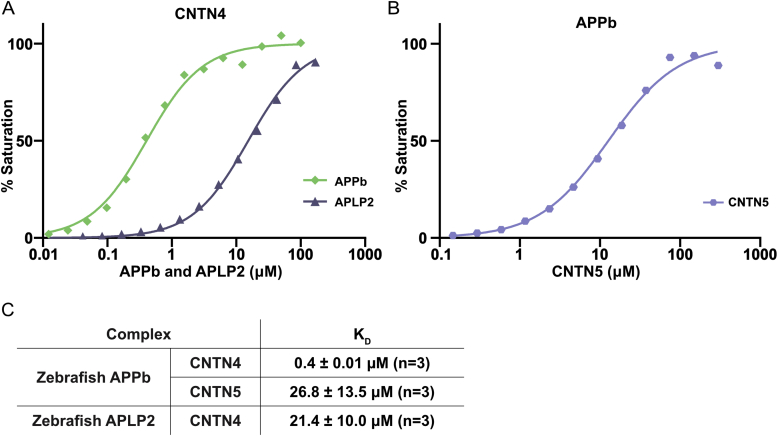
**Characterization of interactions between zebrafish APPb, APLP2, CNTN4, and CNTN5 by bio-layer interferometry.***A*, the FN1-FN3 domains of zebrafish CNTN4 were biotinylated in a 1:1 M ratio, immobilized onto streptavidin sensors, and titrated against varying concentrations of the E1 domains of zebrafish APPb or APLP2. *B*, the E1 domain of APPb was biotinylated in a 1:1 M ratio, immobilized onto streptavidin sensors, and titrated against varying concentrations of the FN1-FN3 region of zebrafish CNTN5. The plots were normalized to the maximal response and one representative experiment for each series is shown in panels *A* and *B*. *C*, summary of the affinities measured for three biological replicates.

We then repeated these experiments using the E1 domain human APLP1 *versus* the FN1–FN3 regions of CNTN3–5 (Fig. 3*B*). In this case, we also found that APLP1 appeared to interact more strongly with CNTN4 than it does with CNTN3 or CNTN5 although the differences in *K*_*D*_ values were less pronounced than in the case of APP. Furthermore, we confirmed the interaction between CNTN4, CNTN5, and APLP2 that we first detected in our extracellular binding assay (Figs. 2*D* and 3*C*). Indeed, human APLP2 interacts with CNTN4 with an affinity of 6.3 μM, which indicates weaker binding compared with CNTN4–APP or CNTN4–APLP1 pairs. The affinity between CNTN5 and APLP2 is also weaker than for the CNTN5–APP pair at ∼38 μM. Finally, we also used BLI to characterize the association between zebrafish APPb, APLP2, CNTN4, and CNTN5 (Fig. S4). The E1 domains of zebrafish APPb and APLP2 are, respectively, 82% and 78% identical to their human homologs, whereas the FN2 domains of zebrafish CNTN4 and CNTN5 share 70% and 76% amino acid sequence identity with the corresponding human domains (Figs. S2 and S3). The results mirror those obtained with the human and mouse proteins: the affinity of APPb for CNTN4 (*K*_*D*_ = 0.40 μM) appeared higher than for APLP2 (*K*_*D*_ = 21.4 μM), whereas CNTN5 bound APPb less strongly than does CNTN4 (*K*_*D*_ = 26.8 μM).

The affinities we measured by BLI are substantially weaker than the previously reported ones for the binding between APP and APLP1 with CNTN3–5 (17–35 nM, (9)). However, these published results were obtained using CNTN ectodomains fused to IgG Fc as well as placental alkaline phosphatase fusions of APP and APLP1, which are dimeric. Thus, we believe the differences in affinities reflect differences in the oligomeric states of the proteins being analyzed. Overall, our binding assays suggest that CNTN4 and CNTN5 associate with all three members of the amyloid family, whereas CNTN3 only binds to APP and APLP1. This work also indicates that among CNTN family members, CNTN4 associates more strongly with amyloid family members than CNTN3 or CNTN5. However, the extent to which these differences in affinities between soluble fragments of CNTNs and amyloid might translate into distinct physiological roles for CNTN–amyloid pairs is unclear because these interactions occur between proteins anchored to the cell surface as they associate into a coreceptor complex (4).

### APP associates with CNTN4 through a small interface

**Figure 4:**
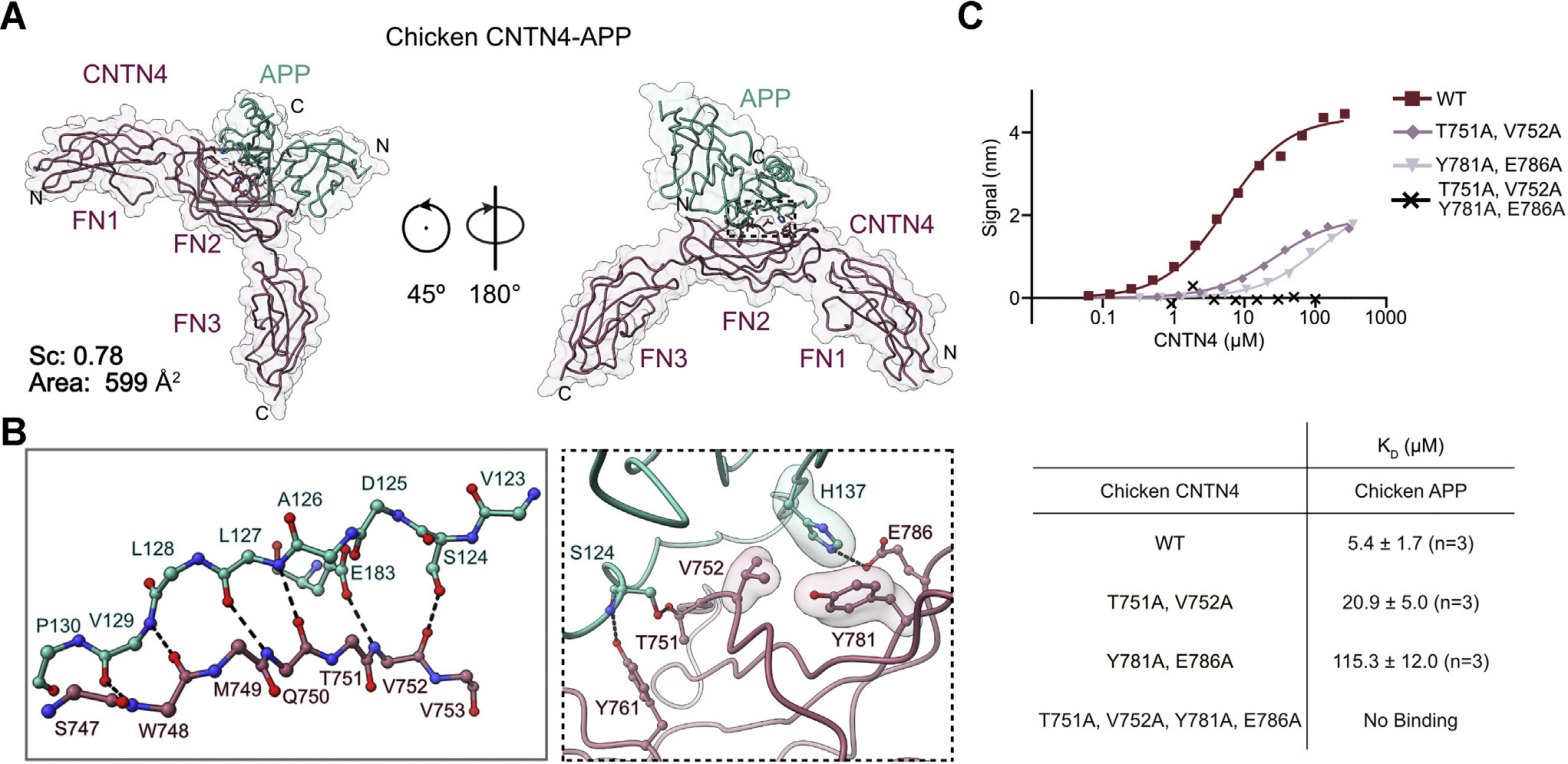
**Structural basis for interactions between APP and CNTN4.***A*, the crystal structure of chicken APP(E1) (*green*) bound to CNTN4(FN1–FN3) (*purple*) is shown in a coil representation superimposed with a translucent surface. The letters N and C indicate the N and C termini, respectively. The interface area occludes 599 Å^2^ of surface area, whereas the shape complementarity (Sc) statistic is consistent with protease–inhibitor complexes (39). *B*, detailed views of the interfaces boxed in *A* showing hydrogen bonding interactions between antiparallel β-strands in CNTN4 and APP. Most contacts involve main-chain hydrogen bonding interactions although the side-chain carboxylate group of E183 and the hydroxyl group of S124 form hydrogen bonds with the nitrogen and oxygen atoms of V752 in CNTN4, respectively. Noninteracting side-chain atoms are not shown for clarity. *Dashed lines* indicate potential hydrogen bonds or salt bridges, whereas translucent surfaces highlight residues involved in packing interactions. *C*, analysis of the chicken CNTN4–APP interface by site-directed mutagenesis. The biotinylated E1 domain of chicken APP was immobilized onto streptavidin sensors and titrated against varying concentrations of the FN1–FN3 region of chicken CNTN4 proteins. One representative experiment for each series is shown in the *top panel*, whereas a summary of the affinities measured for at least three biological replicates is shown below the binding curves. APP, amyloid precursor protein; CNTN4, contactin 4; FN1, first FN repeat; FN3, third FN repeat.

**Supplemental Figure S5:**
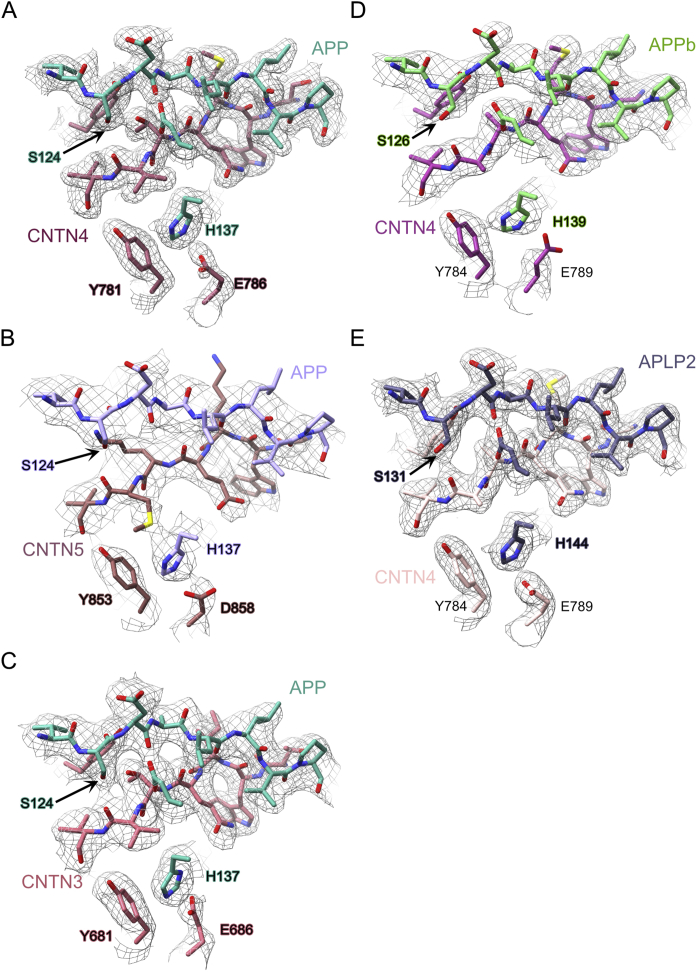
**Representative electron density maps at the interface between members of the CNTN and amyloid families.***A*, 2mFo-DFc electron density map for the chicken CNTN4-APP complex at 2.05 Å is contoured at 1σ. *B*, 2mFo-DFc electron density map for the mouse CNTN5-APP complex at 3.50 Å is contoured at 1σ. *C*, 2mFo-DFc electron density map for the chicken CNTN3-APP fusion protein at 2.75 Å is contoured at 1σ. *D*, 2mFo-DFc electron density map for the zebrafish CNTN4-APPb complex at 2.93 Å is contoured at 1σ. *E*, 2mFo-DFc electron density map for the zebrafish CNTN4-APLP2 at 2.50 Å is contoured at 1σ.

We first sought to gain structural insights into CNTN–amyloid interactions by focusing on the CNTN4–APP complex. Because human APP(E1) seemed prone to aggregation, we attempted crystallization of the chicken and mouse complexes. The amino acid sequences of APP and CNTN4 are very well conserved between the human, mouse, and chicken orthologs. In particular, the E1 domains of chicken and mouse APP are 93% and 97% identical to human APP(E1), respectively, whereas chicken and mouse CNTN4(FN1–FN3) are 85% and 96% identical to human CNTN4(FN1–FN3), respectively (Figs. S2 and S3). Although both the chicken and mouse complexes crystallized, only the former yielded crystals that diffracted well enough to allow structure determination (Table 1). The structure of the chicken CNTN4–APP complex was solved using molecular replacement with the FN1–FN3 region of mouse CNTN4 (Protein Data Bank [PDB] ID: 5E4S) and the E1 domain of mouse APP (PDB ID: 3KTM) as search models and refined to 2.05 Å (*R*_work_/*R*_free_ = 0.183/0.225, Figs. 4 and S5*A*). Overall, there is little change in the E1 domain of chicken APP compared with the published E1 domain of mouse APP ((36), RMSD of 0.475 Å over 155 Cα residues), which is not surprising given the 94% amino acid identity between the two proteins. The FN1–FN3 region of chicken CNTN4 is characterized by a sharp bend between the FN2 and FN3 domains that appears to be a hallmark of vertebrate CNTNs (34). The binding site involves the second FN repeat of CNTN4 and the copper-binding domain of APP, though it does not overlap with residues involved in copper binding (Figs. 4*A* and S2) (37, 38). The complex interface occludes only ∼600 Å^2^ of surface area, but with a high shape complementarity coefficient of 0.78, which is consistent with values calculated for trypsin–trypsin-inhibitor structures (39). The interface is stabilized by a network of main chain–main chain hydrogen bonds between two antiparallel β-strands found in APP (L127–P130) and in CNTN4 (W748–Q750), respectively (Fig. 4*B*). In addition, the side-chain oxygen atom of S124 in APP forms a hydrogen bond with T751 in CNTN4, whereas its main chain oxygen atom forms a hydrogen bond with the side chain of Y761. Finally, H137 is nestled against V752 and Y781 in CNTN4, whereas its imidazole ring interacts with the carboxylate group of E786 (Fig. 4*B*).

**Table 1:** Data collection and refinement statistics

Data set	Chicken CNTN3–APP	Chicken CNTN4–APP	Zebrafish CNTN4–APPb	Zebrafish CNTN4–APLP2	Chicken CNTN4(FN1–FN3) (T751A, V752A, Y781A, and E786A)	Zebrafish CNTN4(FN1–FN3)	Mouse CNTN5–APP
Data collection							
Beamline	APS 22-ID	APS 22-ID	APS 22-ID	APS 22-ID	APS 22-BM	APS 22-ID	APS 22-BM
Wavelength (Å)	1.00	1.00	1.00	1.00	1.00	1.00	1.00
Number of unique reflections	30,550 (4407)	82,428 (4511)	13,383 (2111)	20,199 (2266)	13,597 (1014)	28,351 (1791)	17,993 (4233)
Resolution (Å)	29.87–2.75	64.14–2.05	89.77–2.93	49.96–2.50	50.00–3.20	36.916–1.87	74.79–3.50
Space group	P2_1_2_1_2_1_	I2	P2_1_2_1_2_1_	P2_1_	P2_1_2_1_2_1_	P2_1_2_1_2_1_	H3_2_
Unit cell							
*a*, *b*, *c* (Å)	69.67, 94.50, 174.15	128.45, 65.51, 158.29	74.98, 88.19, 89.77	61.66, 44.98, 99.83	70.94, 91.20, 124.33	44.70, 56.88, 130.94	137.01, 137.01, 385.37
*α*, *β*, *γ* (º)	90.00, 90.00, 90.00	90.00, 92.92, 90.00	90.00, 90.00, 90.00	90.00, 95.84, 90.00	90.0, 90.0, 90.0	90.00, 90.00, 90.00	90.00, 90.00, 90.00
*R*_merge_	0.179 (0.699)	0.101 (0.772)	0.165 (1.163)	0.09 (0.597)	0.193 (0.767)	0.129 (0.268)	0.216 (1.323)
*R*_pim_	0.073 (0.275)	0.044 (0.326)	0.053 (0.384)	0.035 (0.243)	0.058 (0.254)	0.037 (0.075)	0.073 (0.444)
Completeness (%)	99.9 (100.0)	99.7 (100.0)	100.0 (100.0)	100.00 (99.9)	95.1 (71.7)	100.0 (100.0)	100.0 (100.0)
Redundancy	6.9 (7.3)	6.4 (6.5)	10.4 (10.0)	7.4 (7.0)	11.3 (8.3)	13.2 (13.9)	9.7 (9.8)
I/σI	7.8 (2.9)	10.0 (2.3)	9.8 (2.0)	15.6 (3.9)	12.0 (1.9)	12.7 (8.5)	8.8 (1.9)
CC_1/2_	0.987 (0.891)	0.995 (0.805)	0.991 (0.697)	0.998 (0.909)	0.980 (0.846)	0.994 (0.978)	0.983 (0.728)
Refinement							
PDB code	7MRM	7MRK	7MRS	7MQY	7MRQ	7MRO	7MRN
Number of protein chains in asymmetric unit	4	4	2	2	1	1	4
Resolution (Å)	2.75	2.05	2.93	2.46	3.20	1.87	3.50
Reflections (test)	30,480 (2968)	80,010 (1995)	12,777 (1132)	19,739 (1840)	13,415 (1350)	28,274 (2779)	17,982 (1799)
*R*_work_/*R*_free_	0.193/0.231	0.183/0.225	0.240/0.283	0.189/0.221	0.221/0.261	0.174/0.216	0.243/0.291
Number of atoms	5827	6837	2831	2957	2273	2716	6326
Protein	5668	6322	2814	2837	2273	2322	6326
Ligand	20	7	—	—	—	—	—
Water	139	508	17	120	—	384	—
RMSD							
Ideal bonds (Å)	0.003	0.003	0.003	0.004	0.005	0.006	0.002
Ideal angles (°)	0.50	0.60	0.59	0.68	0.82	0.85	0.49
Average *B*-factors (Å^2^)	55.5	49.0	71.8	51.7	132.6	24.1	136.01
Protein	56.0	48.9	71.9	51.9	132.6	22.9	136.01
Ligand	88.4	58.7	—	—	—	—	—
Water	46.9	50.3	62.7	46.6	—	31.3	—
Ramachandran statistics							
Favored (%)	97.20	97.50	97.21	98.07	97.95	97.69	96.85
Allowed (%)	2.80	2.50	2.79	1.93	2.05	2.31	3.15
Rotamer outlier (%)	0.0	0.0	0.65	0.32	0.0	0.40	0.28

Values in parentheses apply to the high-resolution shell.

**Supplemental Figure S6:**
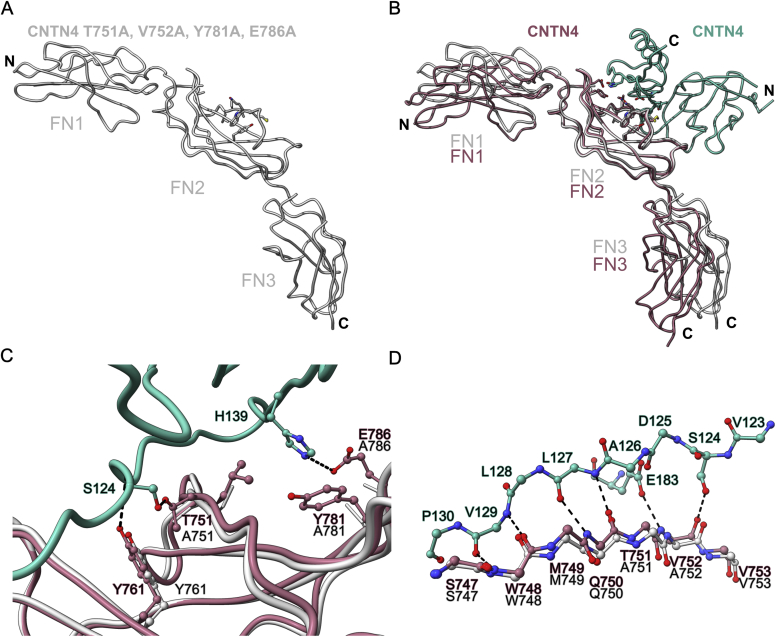
**Introducing the mutations T751A, V752A, Y781A, and E786A in chicken CNTN4 does not induce any significant change in the structure of CNTN4.***A*, the structure of chicken CNTN4(FN1-FN3) including the point mutations T751A, V752A, Y781A, E786A (TVYE-AAAA) refined to 3.20 Å resolution (R_work_/R_free_ = 0.217/0.258) is shown here in coil representation. *B*, superimposition of the quadruple alanine mutant of chicken CNTN4(FN1-FN3) (silver) over wild type chicken CNTN4(FN1-FN3) bound to chicken APP-CNTN4. The RMSD between 100 Cα pairs in the second FN domain is 0.50 Å. *C*, detailed view of side chain-side chain interactions at the CNTN4-APP interface superimposed on the structure of the CNTN4(FN1-FN3) mutant. Dashed lines indicate potential hydrogen bonds or salt bridges. *D*, hydrogen bonding network between antiparallel β-strands in CNTN4 and APP. Residues in the mutant CNTN4 structure are shown in silver. Non interacting side chains atoms are omitted for clarity.

We designed three variants of chicken CNTN4 by changing interface residues to alanine to validate our structural results. Residues T751/V752 or Y781/E786 were substituted with alanine in the first two mutants, whereas all four residues were mutated to alanine in the third. Binding assays using BLI were carried out as described previously with the biotinylated E1 domain of chicken APP immobilized on streptavidin sensors. Introducing alanine residues at positions 751 and 752 in CNTN4 resulted in a fourfold-reduced affinity for APP (Fig. 4*C*). Residues Y781 and E786 interact with H137 in APP, and changing these two residues to alanine led to a 20-fold weaker binding (*K*_*D*_ = 115 μM *versus* 5.4 μM). This finding is consistent with the fact that changing H137 to alanine impairs binding of APP to the CNTN4 homolog CNTN3 (10). Finally, introducing alanine residues at positions T751, V752, Y781, and E786 results in a protein that does not bind to immobilized APP (Fig. 4*C*). Importantly, this difference in binding affinity is not because of any gross structural change brought about by the introduction of alanine residues as evidenced by comparing the structure of chicken CNTN4 with a mutant that includes all four alanine mutations (Fig. S6). Overall, these binding analyses indicate that the interface identified between the FN2 of CNTN4 and the copper-binding region of APP represents likely the arrangement of these two receptors on the surface of cells.

### The arrangement of the CNTN–APP complexes is conserved

**Figure 5:**
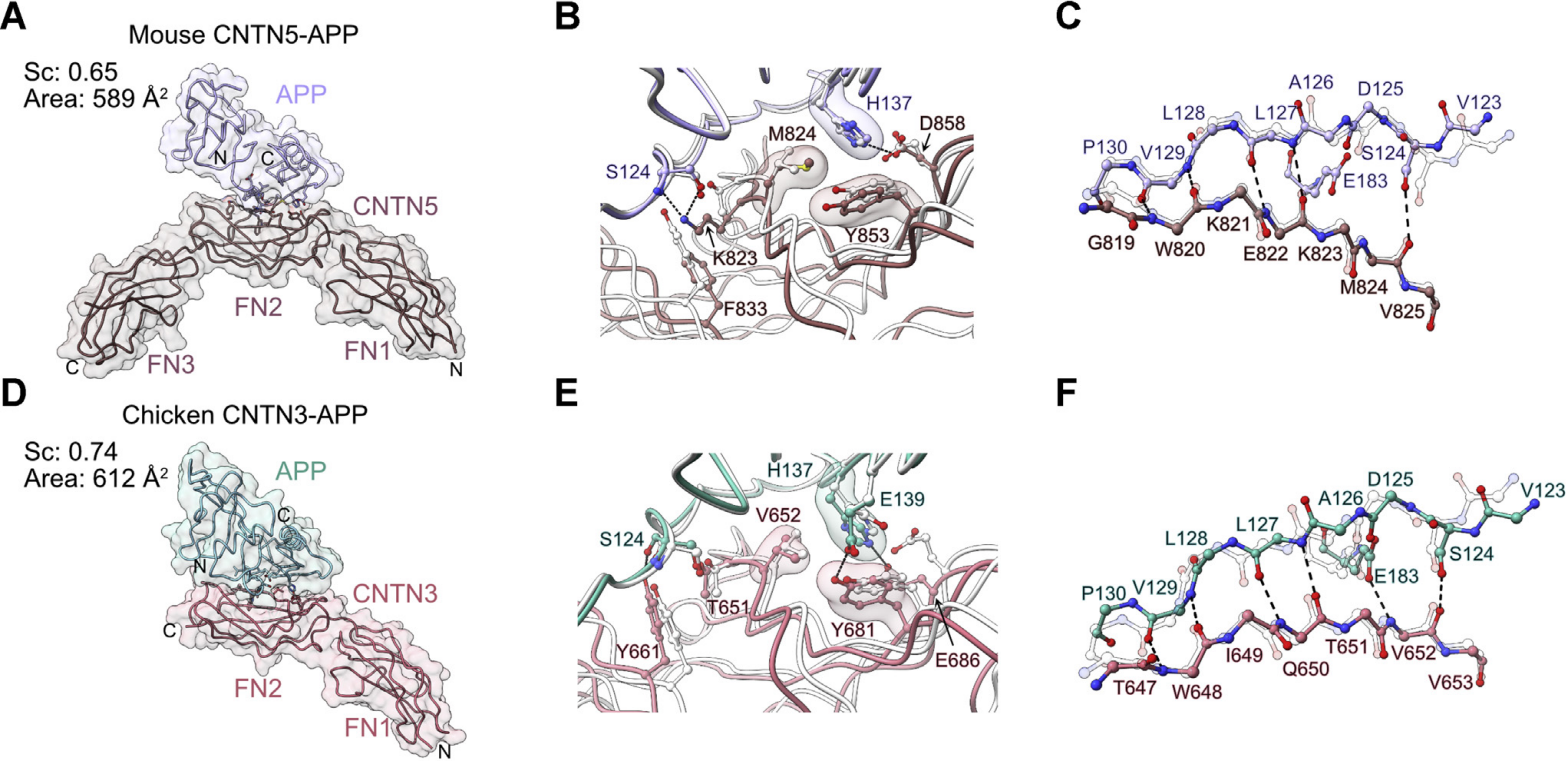
**Crystal structures of the mouse CNTN5–APP complex and chicken CNTN3–APP fusion protein.***A*, the crystal structure of mouse APP(E1) bound to the FN1–FN3 region of mouse CNTN5 is shown in coil representation along with a translucent surface. *B*, detailed view of side chain–side chain interactions at the CNTN5–APP interface. *Dashed lines* indicate potential hydrogen bonds or salt bridges, whereas translucent surfaces highlight residues involved in packing interactions. *C*, main chain–main chain and side chain–main chain hydrogen bonding network, indicated by *dashed lines*, between antiparallel β-strands in CNTN5 and APP. Side chains not involved in contacts are removed for clarity. In *B* and *C*, corresponding residues at the CNTN4–APP interface are superimposed and shown in *white*. The structures were superimposed using the copper-binding domains of APP (RMSD of 0.46 Å over 69 Cα pairs). *D*, the crystal structure of the fusion protein between the FN1–FN2 region of chicken CNTN3 fused to the E1 domain of chicken APP is shown in coil and surface representations. *E*, detailed view of side chain–side chain interactions at the CNTN3–APP interface. *Dashed lines* indicate potential hydrogen bonds or salt bridges, whereas translucent surfaces highlight residues involved in packing interactions. *F*, main chain–main chain and side chain–main chain hydrogen bonding network, indicated by *dashed lines*, between antiparallel β-strands in CNTN3 and APP. Side chains not involved in contacts are removed for clarity. In *E* and *F*, corresponding residues at the CNTN4–APP interface are superimposed and shown in *white*. The structures were superimposed using the copper-binding domains of APP (RMSD of 0.25 Å over 69 Cα pairs). APP, amyloid precursor protein; CNTN3, contactin 3; CNTN5, contactin 5; FN1, first FN repeat; FN2, second FN repeat; FN3, third FN repeat.

We next asked whether differences exist in the details of the interactions between additional CNTN and amyloid family members. We thus initiated crystallization trials with the E1 domain of mouse APP and the FN1–FN3 regions of mouse CNTN3 and CNTN5 but only succeeded in obtaining a crystal structure of the CNTN5–APP complex at moderate resolution (3.5 Å, *R*_work_/*R*_free_ = 0.243/0.291, Figs. 5*A* and S5*B*). In addition, complexes formed by chicken CNTN3(FN1–FN3) and APP(E1) also proved recalcitrant to crystallization. To resolve this issue, we designed a fusion protein of CNTN3 and APP, speculating that it might yield suitable crystals. We thus fused chicken CNTN3 to APP by inserting a GGGSGGGS linker between the C terminus of the FN2 repeat of CNTN3 and the N terminus of the E1 domain of chicken APP. This chimeric protein crystallized readily, and we refined the structure of the CNTN3–APP fusion to 2.75 Å (*R*_work_/*R*_free_ = 0.197/0.231, Figs. 5*D* and S5*C*). No electron density for the linker could be observed in the maps.

Overall, the binding sites identified in the structures of the CNTN5–APP complex and the CNTN3–APP fusion protein are similar to the one identified in CNTN4–APP structure (Fig. 5, *B*, *C*, *E*, and *F*). As is the case in CNTN4–APP, the interfaces include hydrogen bonding interactions between main chain atoms between two β-strands in APP and CNTN3–5 (Fig. 5, *B* and *E*). Residue H137 in APP contacts Y681 in CNTN3 (Y853 in CNTN5), and its side chain forms a hydrogen bond with the carboxylate group of E686 (D858 in CNTN5) (Fig. 5, *C* and *F*). These contacts are analogous to the H137–E786 interaction found at the interface between chicken APP with CNTN4 (Fig. 4*B*). H137 also packs against V652 in CNTN3, as is observed at the CNTN4–APP interface. As mentioned previously, these findings are in line with experiments demonstrating that the H137A mutation impairs the interaction between mouse CNTN3 and mouse APP (10). In the CNTN3–APP structure, we observed a hydrogen bond between the hydroxyl group of Y681, which hydrogen bonds with E139 (Fig. 5*E*). This feature is absent in the CNTN5–APP complex (Fig. 5*B*) but found in zebrafish CNTN4–APPb and CNTN4–APLP2 structures as indicated later. At the CNTN3–APP interface, residue T651 forms a hydrogen bond with S124. This contact is identical to the T751–S124 hydrogen bond observed in the CNTN4–APP complex. Finally, even though residues T751 and V752 in chicken CNTN4 are replaced by K823 and M824 in mouse CNTN5, these lysine and arginine residues still form a hydrogen bond with S124 and van der Waals interactions with H137, respectively (Fig. 5*B*). Thus, the sum of these structural analyses indicates that the topology of complexes between APP and CNTN3–5 is conserved.

### The interfaces between zebrafish APPb, APLP2, and CNTN4 share features found in other CNTN–APP complexes

**Figure 6:**
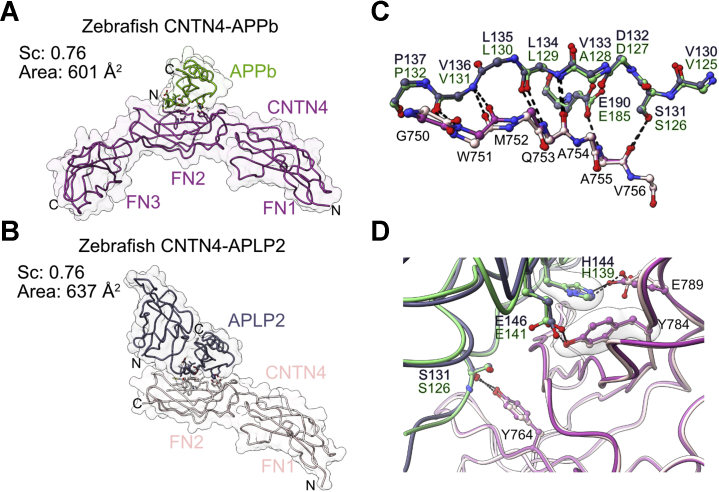
**Crystal structures of zebrafish CNTN4 bound to APPb and APLP2.***A*, crystal structure of the copper-binding domain of zebrafish APPb in complex with CNTN4(FN1–FN3). *B*, crystal structure of the fusion protein between zebrafish CNTN4(FN1–FN2) and the E1 domain of APLP2. In *A* and *B*, the structures are shown in coil and surface representations, whereas the letters N and C indicate the N and C termini, respectively. *C*, detailed view of side chain–side chain interactions at the CNTN4–APPb and CNTN4–APLP2 interfaces. *Dashed lines* indicate potential hydrogen bonds or salt bridges, whereas translucent surfaces highlight residues involved in packing interactions. *D*, main-chain hydrogen bonding network, indicated by *dashed lines*, between antiparallel β-strands in CNTN4 and APPb–APLP2. The structures were superimposed using the copper-binding domains of APPb and APLP2 (RMSD of 0.57 Å over 62 Cα pairs). APLP2, amyloid beta precursor like protein 2; APP, amyloid precursor protein; CNTN4, contactin 4; FN1, first FN repeat; FN2, second FN repeat; FN3, third FN repeat.

Introducing the T751A and V752A mutations in chicken CNTN4 reduced its affinity for APP by fourfold (Fig. 4*E*), so we found it intriguing that these changes occur naturally in zebrafish CNTN4 (Fig. S3). We thus decided to gain structural insights into the zebrafish complexes to determine the extent to which details of interactions differed when compared with chicken CNTN4–APP. As explained previously, human APP appears more closely related to APPb than APPa (40), so we crystallized the E1 domain of APPb and the FN1–FN3 region of zebrafish CNTN4. Surprisingly, subsequent structural analysis indicated that a proteolytic event had likely released the growth factor–like domain of APPb so that only its copper-binding domain bound to CNTN4 (2.93 Å, *R*_work_/*R*_free_ = 0.241/0.281, Figs. 6*A* and S5*D*). Because of the difficulty we encountered in obtaining the CNTN4–APPb crystals, we followed the strategy we employed for chicken CNTN3–APP crystals and generated a fusion protein between the FN1–FN2 region of zebrafish CNTN4 and the E1 domain of zebrafish APLP2. The structure of this chimeric protein was refined to 2.46 Å (*R*_work_/*R*_free_ = 0.189/0.221, Figs. 6*B* and S5*E*).

**Supplemental Figure S7:**
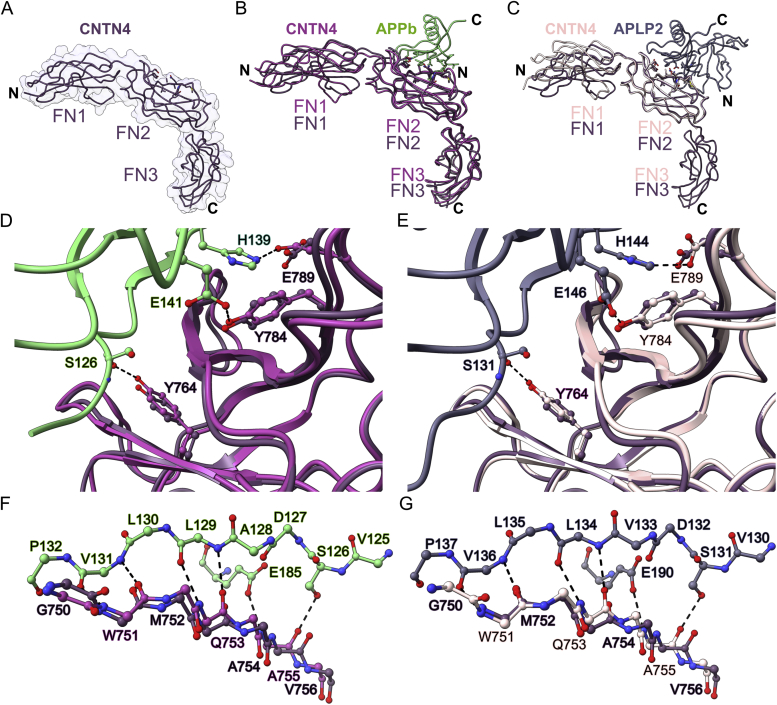
**Comparison of the zebrafish CNTN4(FN1-FN3) structure with those of CNTN4 bound to APPb and APLP2.***A*, the crystal structure of zebrafish CNTN4(FN1-FN3) was refined to 1.87-Å resolution (R_work_/R_free_ = 0.174/0.216) and is shown here in coil and surface representations. *B*, the crystal structure of zebrafish CNTN4(FN1-FN3) is colored purple and superimposed onto the crystal of CNTN4-APPb (colored magenta and green). The FN2 domains of CNTN4 were used for the superimposition (RMSD of 0.63 Å over 101 Cα pairs). *C*, the crystal structure of zebrafish CNTN4(FN1-FN3) is colored purple and superimposed onto the crystal of CNTN4-APPb (colored light pink and slate). The FN2 domains of CNTN4 were used for the superimposition (RMSD of 0.47 Å over 99 Cα pairs). *D*, detailed view of side chain-side chain interactions at the CNTN4-APPb interface superimposed on the structure of the CNTN4(FN1-FN3). Dashed lines indicate potential hydrogen bonds or salt bridges. *E*, detailed view of side chain-side chain interactions at the CNTN4-APLP2 interface superimposed on the structure of the CNTN4(FN1-FN3). Dashed lines indicate potential hydrogen bonds or salt bridges. *F*, hydrogen bonding network between antiparallel β-strands in CNTN4 (magenta) and APPb (green). Residues in the unbound CNTN4 structure are shown in purple. Non interacting side chains atoms are omitted for clarity. *G*, hydrogen bonding network between antiparallel β-strands in CNTN4 (light pink) and APLP2 (slate). Residues in the unbound CNTN4 structure are shown in purple. Non interacting side chains atoms are omitted for clarity.

**Supplemental Figure S8:**
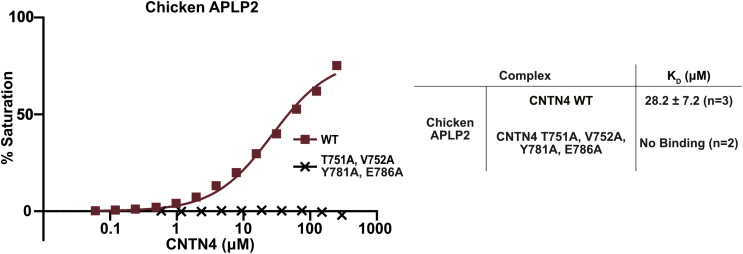
**Biochemical analysis of chicken APLP2 binding to CNTN4.** The biotinylated E1 domain of chicken APLP2 was immobilized onto streptavidin sensors and incubated varying concentrations of the FN1-FN3 region of chicken CNTN4 proteins (WT and alanine mutant). One representative experiment for each series is shown while a summary of the affinities measured for at least two biological replicates are shown on the right.

Overall, the CNTN4–APPb and CNTN4–APLP2 interfaces display many of the same features found in the cocrystals of chicken CNTN4 and APP (Figs. 4*B* and 6, *C* and *D*). As is the case in previous CNTN–APP complex structures (Figs. 4 and 5), the bulk of the interactions includes main chain–main chain hydrogen bonds between two β-strands: W751–Q753 in CNTN4 and L129–P132 in APPb or L134–P137 in APLP2 (Fig. 6*C*). As is the case in the CNTN3–APP and CNTN4–APP interfaces, the carbonyl oxygen atom of S126 in APPb (S131 in APLP2) forms a hydrogen bond with the hydroxyl group of Y764 (Fig. 6*D*). Furthermore, CNTN4 residues Y784 and E789 form hydrogen bonds with E141 and H139, respectively, in APPb, and with E146 and H144, respectively, in APLP2 (Fig. 6*D*). These contacts are absent in the chicken CNTN4–APP and mouse CNTN5–APP complexes. Finally, complex formation does not involve any substantial structural change in CNTN4, since the structure of the unbound FN2 domain in CNTN4 matches those of amyloid-bound CNTN4 closely (Fig. S7). Overall, the sum of this crystallographic work indicates that the CNTN4–APLP2 interface in zebrafish is essentially identical to the CNTN4–APPb interface in zebrafish and the CNTN4–APP interface in chicken. Consistent with these crystallographic results, mutations of residues T751, V752, Y781, and E786 to alanine in chicken CNTN4 that prevented binding to CNTN4 also abolished its binding to APLP2 (Figs. 4*C* and S8).

### A molecular basis for CNTN–amyloid specificity

**Figure 7:**
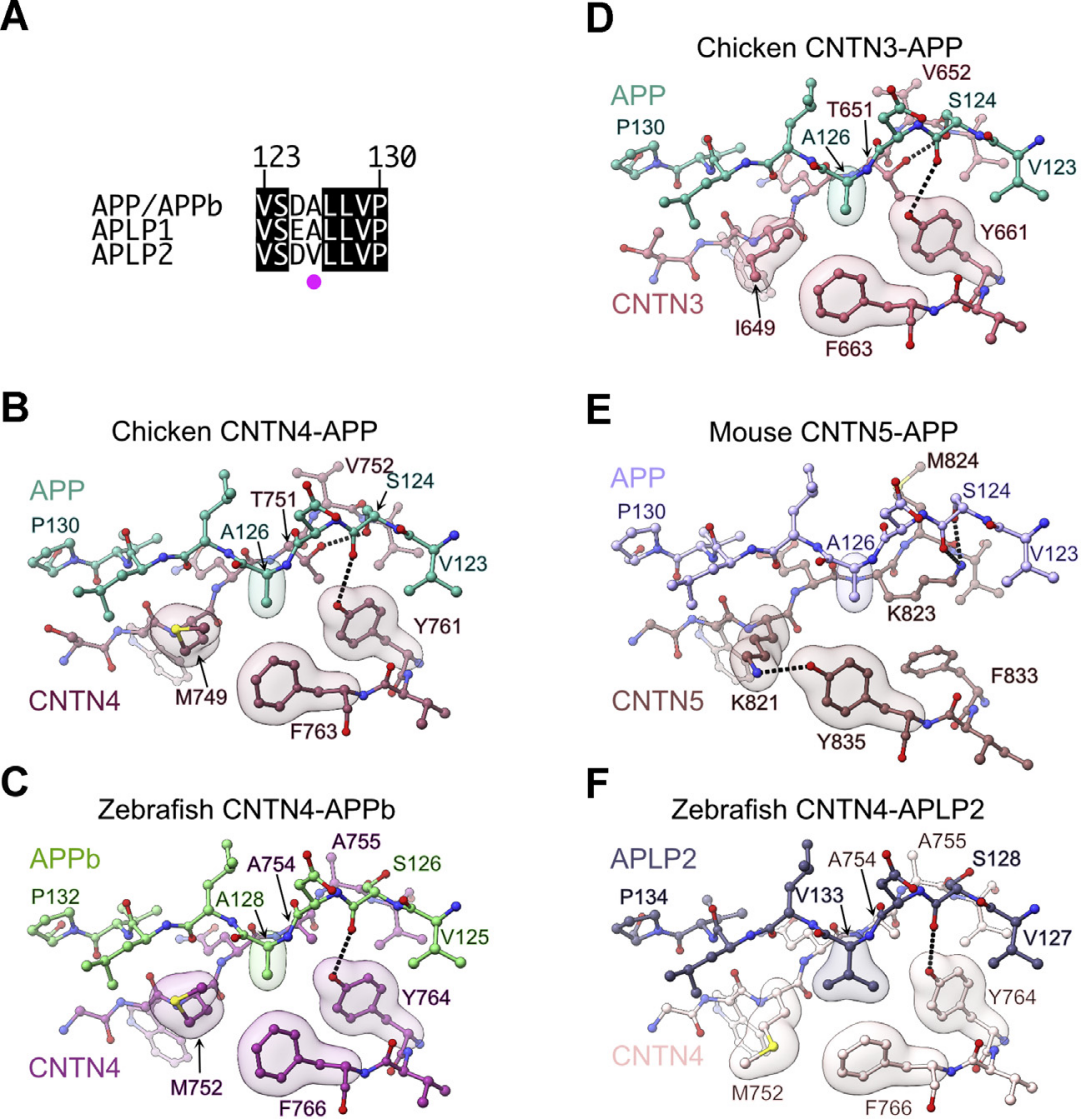
**Distinct contacts at CNTN–amyloid interfaces.***A*, sequence alignment of APP/APPb, APLP1, and APLP2. In the limited segments shown here, the sequences of human, mouse, chicken APP, and zebrafish APPb are identical. Similarly, the sequences of human, mouse, and zebrafish APLP1 are identical, and the sequences of human, mouse, chicken, and zebrafish APLP2 are identical. Strictly conserved residues are shown in *black*. The *magenta dot* indicates the position of A126, which is conserved in APP and APLP1, but replaced by a valine in APLP2. The numbering above the sequences corresponds to amino acid positions in chicken APP, which is identical in mouse and human APP. A more extensive alignment is shown in Fig. S2. *B*, detailed view of the chicken CNTN4–APP interface with translucent surfaces highlighting APP residue A126 nestled against M749, Y761, and Y763 in CNTN4. *Dashed lines* indicate hydrogen bonds between T751, Y761, and S124. *C*, detailed view of the zebrafish CNTN4–APPb interface highlighting interactions between APPb residue A128 and CNTN4 residues M752, Y764, and F766. Note that compared with the chicken sequence, the sequence of chicken CNTN4 is offset by three amino acids. *D*, detailed view of the chicken CNTN3–APP interface. Here, A126 contacts I649, Y661, and F663. *Dashed lines* indicate hydrogen bonds between T651, Y661, and APP residue S124. *E*, detailed view of the mouse CNTN5–APP interface. Here, APP residue A126 contacts the aliphatic portion of K821 and Y835 but not F833. *Dashed lines* indicate hydrogen bonds between K821 and Y835 in CNTN5 and between K823 in CNTN5 and S124 in APP. *F*, detailed view of the zebrafish CNTN4–APLP2 interface. The alanine residue found in APP is replaced by a valine in APLP2 (V133), which contacts M752, Y764, and F766. The side chain of M752 has swung away from the position it occupies in the chicken CNTN4–APP and zebrafish CNTN4–APPb complexes. APLP2, amyloid beta precursor like protein 2; APP, amyloid precursor protein; CNTN, contactin.

Following our structural analyses of CNTN–amyloid interfaces, we wanted to rationalize the following observations: (i) APP associates more strongly to CNTN4 than it does to CNTN3 or CNTN5 (Fig. 3*A*), (ii) CNTN4 has higher affinity for APP than for APLP2 (Fig. 3, *A* and *C*), and (iii) CNTN3 does not appear to interact to APLP2 (Fig. 2*D*). In our hands, the E1 domains of APP, APLP1, and APLP2 as well as the FN1–FN3 fragments of the CNTN3–5 fragments were monomeric, as judged by size-exclusion chromatography. This observation excluded oligomerization as a potential reason for the distinct binding affinities we measured. We thus inspected the sequences of APP, APLP1, and APLP2 and noticed that all the APP residues at the interface with CNTN4 are conserved in human, mouse, chicken, and zebrafish except A126, which is always replaced by a valine in APLP2 (Figs. 7*A* and S2). In the chicken CNTN4–APP complex, A126 forms van der Waals interactions with the side-chain atoms of M749, Y761, and F763 (Fig. 7*B*). These contacts are conserved in the zebrafish CNTN4–APPb complex (Fig. 7*C*) as well as at the interface between chicken CNTN3 and APP although the methionine residue is replaced by the branched aliphatic residue isoleucine (Fig. 7*D*). Residue M749 is conserved in human, mouse, chicken, and zebrafish CNTN4, whereas the isoleucine residue is conserved in human, mouse, and chicken CNTN3 (Fig. S3). In CNTN5, the arrangement of residues surrounding APP A126 is distinct because the Cξ atom of F833 is located 5.3 Å away from the Cβ of A126, which is too far to mediate a van der Waals interaction (Fig. 7*E*). In this complex, A126 only contacts the aliphatic portion of K821 and the side chain of Y835, which, respectively, replace the methionine and phenylalanine residues that abut A126 in the CNTN4–APP and CNTN4–APPb complexes (Fig. 7*E*). Finally, in APLP2, the alanine residue is replaced by a bulkier Cβ-branched valine, and the side chain of M752 appears to swing away from V133 (Fig. 7*E*). We thus hypothesized that contacts around A126 in APP may account for some of the differences in affinities we measured between CNTN3–5 (Fig. 3).

**Figure 8:**
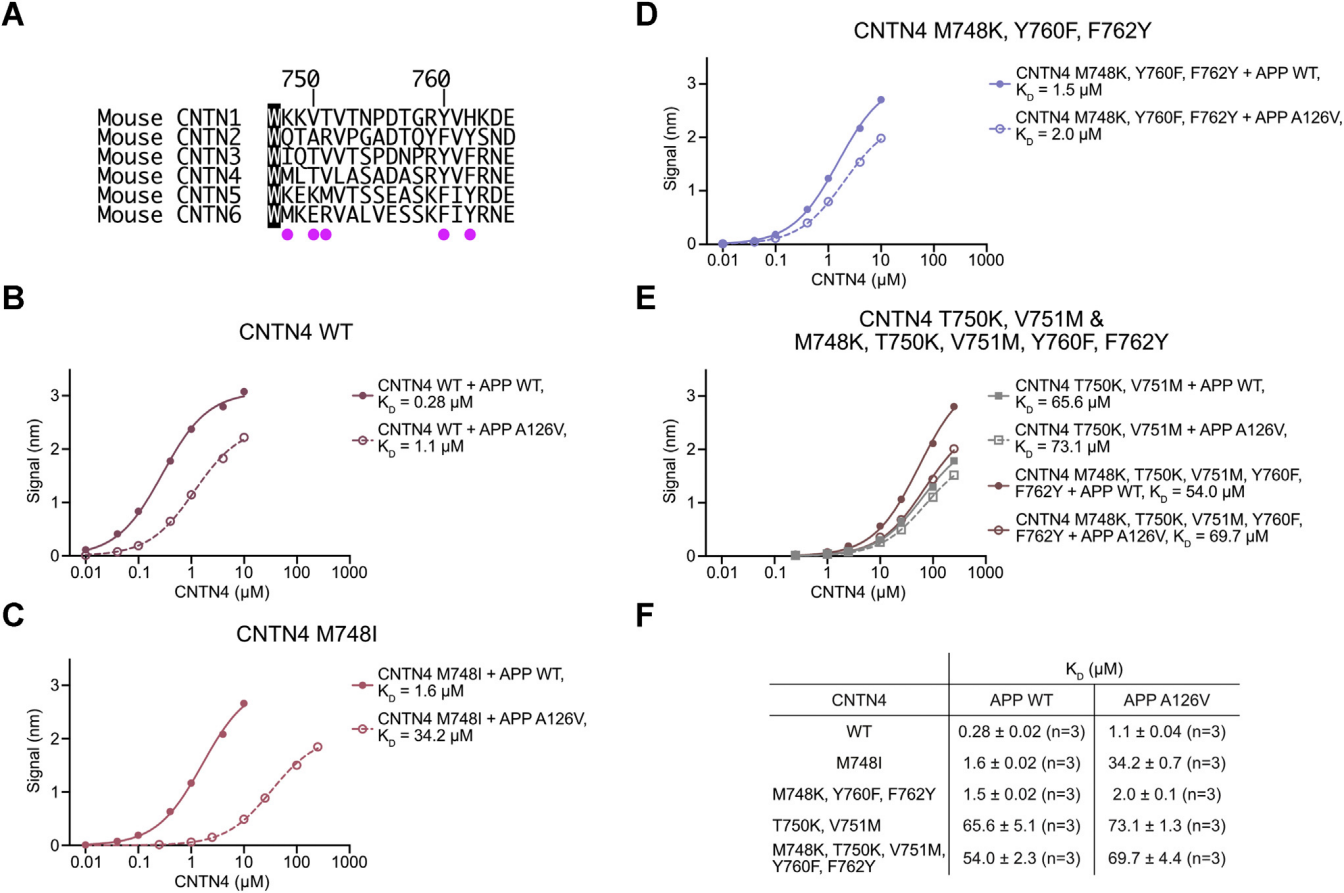
**Binding specificity in CNTN–amyloid interactions.***A*, sequence alignments of mouse CNTN1–6 for a subset of residues at the CNTN–amyloid interface. Identical residues are shown in *black*. *Magenta dots* indicate the positions of amino acids that are mutated in mouse CNTN4 in the experiments shown in *B*–*E*. *B*–*E*, analysis of CNTN4–APP interactions by BLI following mutations at the CNTN4–APP interface. In all experiments, the biotinylated E1 domain of mouse APP or its A126V variant was immobilized onto streptavidin sensors and titrated against varying concentrations of the FN1–FN3 region of mouse CNTN4 proteins. One representative experiment for each experimental series is shown in *B*–*E*. *F*, summary of affinities measured for three biological replicates for each set of proteins. APP, amyloid precursor protein; BLI, biolayer interferometry; CNTN, contactin; FN1, first FN repeat; FN3, third FN repeat.

We used sequence alignments to design variants of mouse APP and mouse CNTN4 (Figs. 7*A* and 8*A*) and measured their interactions using BLI (Fig. 8, *B*–*E*). We started by introducing a valine residue in place of A126 in mouse APP (Fig. 8*B*). Consistent with previous experiments (Fig. 3), mouse CNTN4 bounds to APP with an affinity of 0.28 μM, but introduction of the A126V mutation in APP decreased its affinity for CNTN4 fourfold (1.1 μM *versus* 0.28 μM). The increase in the value of *K*_*D*_ compares well to the increase from 5.4 to 28.2 μM measured between chicken CNTN4–APP and chicken CNTN4–APLP2 (Figs. 4 and S8), respectively. Furthermore, residue A126 is also conserved in APLP1 (Figs. 7*A* and S3), and the affinity between human CNTN4 and APLP2 is decreased by fourfold compared with the affinity between human CNTN4 and APLP1 (Fig. 3). Conversely, the weakening of affinity we measured by introducing the A126V change in mouse APP does not match the 50-fold decrease measured in the case of zebrafish CNTN4 with APPb and APLP2 (Fig. S4). Nevertheless, the results of these assays suggest that contacts around APP residue A126 explain, at least in part, why APLP2 does not bind CNTN4 as strongly as APP does.

Because M749 in CNTN4 is replaced by an isoleucine in CNTN3 (Figs. 7, *B* and *D* and 8*A*), we wondered whether this variation could account for the reduced affinity that APP has for CNTN3. We thus purified a variant of mouse CNTN4(FN1–FN3) that includes that M748I change and measured its binding to mouse APP (Fig. 8*C*). Consistent with the aforementioned results, the M748I mutation decreased the affinity sixfold (1.6 μM *versus* 0.28 μM), suggesting that the presence of the branched isoleucine prevents optimal contacts with APP. Furthermore, in an attempt to understand why CNTN3 does not bind to APLP2, we quantified the affinity between the M748I mutant of CNTN4 and the A126V variant of mouse APP (Fig. 8*C*). In this case, the *K*_*D*_ increased to 34.2 μM, more than a 100-fold increase compared with the *K*_*D*_ between mouse CNTN4 and APP. To be sure, we have not been able to recreate the lack of binding between CNTN3 and APLP2 or measure *K*_*D*_ values between the mutant forms of CNTN4 and APP that would match exactly those measured for CNTN3 and APP. However, the effects of the mutations we have introduced in CNTN4 and APP help rationalize the distinct binding affinities between CNTN3, CNTN4, APP, and APLP2.

We next wondered whether the nature of the contacts surrounding A126 in CNTN5 could also explain why CNTN5 binds APP weakly compared with CNTN4 (Figs. 3*A* and 7*E*). We introduced three changes in mouse CNTN4 in order to recreate the topology observed in CNTN5: Y760F, F762Y, and M748K (Fig. 8*D*). We considered that these more extensive mutations were necessary, as opposed to the sole M748K mutation, because K821 in CNTN5 is held in place through a hydrogen bond with Y835 (Fig. 7*E*). Furthermore, F833 does not contact A126 in the CNTN5–APP complex, so we opted to replace Y760 by a phenylalanine residue. We hypothesized that although these changes might reduce the affinity to APP by limiting contacts with A126, they could also help accommodate the bulkier valine in the A126V mutant of APP. Indeed, the substitutions Y760F, F762Y, and M748K decreased the affinity for APP fivefold, but combining the Y760F, F762Y, and M748K changes in CNTN4 and the A126V mutation in APP only resulted in a sevenfold reduction in affinity (Fig. 8*D*). This trend is similar to what we have observed with CNTN5: it has reduced affinity for APP when compared with CNTN4 (Fig. 3*A*), but it still binds to APLP2, whereas CNTN3 does not (Fig. 2*D*).

Nevertheless, we wondered whether additional changes may explain the significantly weaker affinity that CNTN5 has for APP or APLP2 (Figs. 3*C* and S4). In particular, T751 in chicken CNTN4 forms a hydrogen bond with S124 in APP, whereas V752 packs against H137 (Fig. 4*B*). These residues are conserved in CNTN3, but not in CNTN5, where they are replaced by a lysine and a methionine residue, respectively. However, K823 and M824 mediate the same hydrogen bonding and packing interactions with S124 and H137 as T751 and V752 do (Figs. 4*B* and 5*B*). We found it interesting that larger residues such as lysine and methionine would mediate interactions identical to threonine and valine and wondered whether these changes would shift the respective positions of CNTN5 and APP, thus affecting the “fit” in the complex. Indeed, the shape complementarity coefficient *S*_C_ for CNTN4–APP is 0.78, whereas it is only 0.65 for CNTN5–APP (Figs. 4*A* and 5*A*). To test this hypothesis, we introduced the changes T750K and V751M in mouse CNTN4 (Fig. 8*E*). These two mutations led to more than a 200-fold decrease in affinity when compared with CNTN4 (65.6 μM *versus* 0.28 μM). Interestingly, the decrease in affinity was only 70-fold when we repeated the experiments with the A126V mutant of APP, possibly indicating that the mutations T750K and V751M were not as deleterious in the context of the A126V change. We thus tested a CNTN4 mutant that included mutations that alter contacts around S124 and H137 (T750K and V751M) as well as around A126 (Y760F, F762Y, and M748K). In this case, our results suggest that the additional mutations mitigate the presence of T750K and V751M in the case of APP binding as the *K*_*D*_ decreases from 65.6 to 54.0 μM but have little effect in the case of the A126V APP variant (Fig. 8*E*).

**Figure 9:**
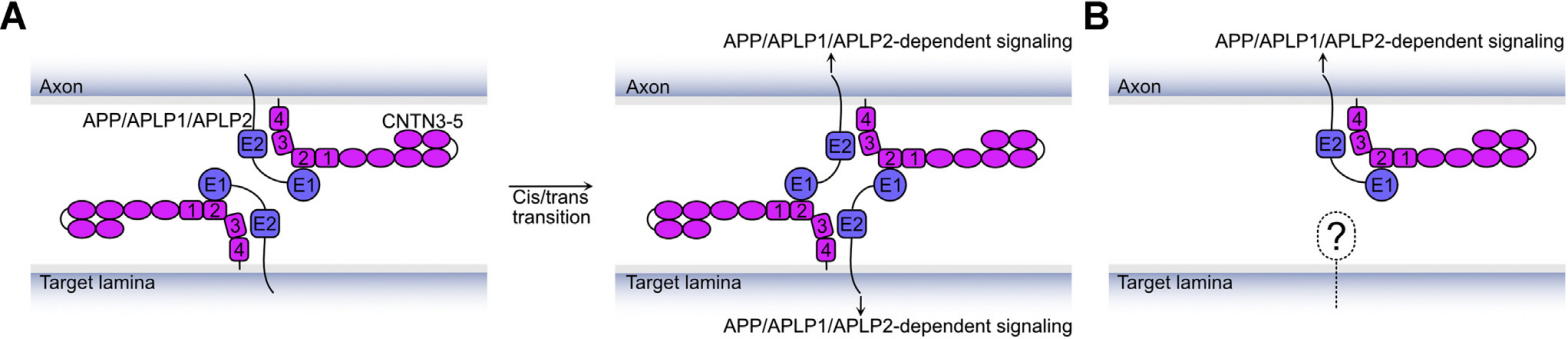
**A model illustrating how a CNTN–amyloid complex would mediate interactions with receptors expressed on specific target cells.***A*, CNTN3–5 form *cis* complexes on axonal surfaces as well as the surfaces of target cells. The flexibility of the linker regions between the E1 and the E2 domains of APP, APLP1, and APLP2 makes it possible to bind to the second FN domain of CNTNs on the same cell or on an apposing cell. Alternatively, a CNTN–amyloid pair on axons could interact with an as of yet unknown partner on the target surface (*B*). APLP1, amyloid beta precursor like protein 1; APLP2, amyloid beta precursor like protein 2; APP, amyloid precursor protein; CNTN, contactin; FN, fibronectin type III repeat.

**Supplemental Figure S9:**
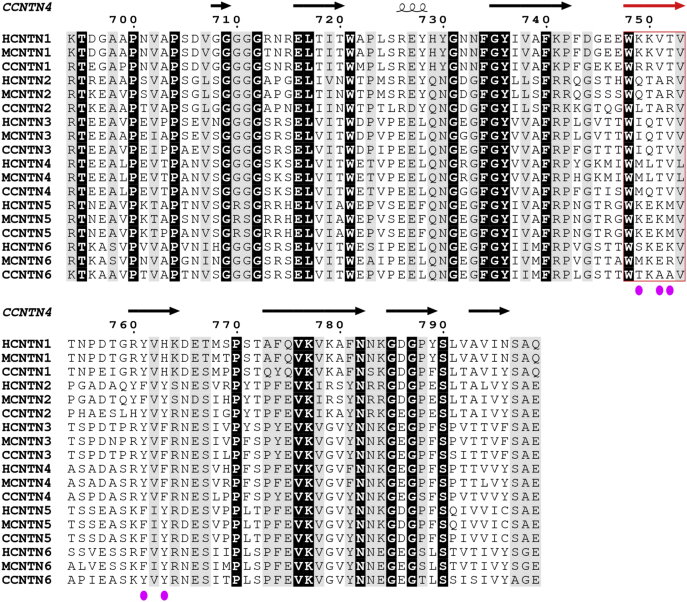
**Alignment of amino acid sequences of FN2 domains from human, mouse, and chicken CNTN1–6.** The secondary structure of chicken CNTN4 is shown above the sequence and the residue numbering corresponds to chicken CNTN4. Identical residues are shown in black, while similar positions are shaded grey. Residues found in a β-strand involved main chain/main chain hydrogen bond interactions with a β-strand in APP and APLP2 are boxed in red. Magenta dots indicate the positions of residues M749, T751, V752, Y761, and F763 (chicken CNTN4 numbering) that appear to determine the specificity between CNTN and amyloid family members.

Taken together, the sum of our mutational analyses indicates that the identities of the CNTN residues that contact the APP amino acids S124, A126, and H137, account, at least in part, for the differences in affinities we measured between APP, APLP2, CNTN3, CNTN4, and CNTN5 (Fig. 3). In that context, we can start to rationalize the absence of interactions between CNTN1, CNTN2, and CNTN6 with amyloid family members (Fig. 2*D*, (9, 10)). Residues T751 and V752 are replaced by valine and threonine, respectively in human, mouse, and chicken CNTN1 (Fig. S9). Furthermore, M749 is replaced by lysine or arginine, whereas Y761 is replaced by a smaller histidine residue. Thus, the sum of these specific changes may explain why CNTN1 does not bind to APP, APLP1, or APLP2. Similarly, T751 is replaced by an alanine in CNTN2, whereas V752 is replaced by a bulkier and positively charged arginine residue, which might not adequately pack against H137. Finally, a similar change can be found in human and mouse CNTN6 where T751 is replaced by a larger glutamate residue and V752 is changed to a lysine or an arginine. These changes are not present in chicken CNTN6, but T751 and V752 are replaced by alanine residues, whereas M749 is changed to threonine, a branched residue. We surmise that sequence variations at positions M749, T751, V752, F761, and F763 partly explain the distinct binding affinities between amyloid and CNTN family members.

## Discussion

Previous work performed in mice suggested that APP and CNTN4 form a coreceptor complex that promotes the arborization of axons from a subpopulation of retinal ganglion cells to a specific region of the mammalian visual system called the nucleus of the optic tract (4). In particular, it was shown that ectopic expression of CNTN4 in axons of ganglion cells that normally do not innervate the nucleus of the optic tract would bias the formation of arbors to this specific area. The conserved arrangement of APP and CNTN4 in the chicken and zebrafish structures we report herein is consistent with the formation of a *cis* CNTN4–APP complex (Fig. 9). Indeed, the N-terminal E1 domain of APP is followed by a linker region of ∼100 amino acids that may provide the flexibility necessary for APP to bind to CNTN4 in a *cis*-orientation, whereas previous structural analyses of CNTN family members suggest that they might lie parallel to the cell surface (34). Because the arrangement observed in the CNTN4–APP complex is conserved in the CNTN3–APP, CNTN5–APP, and CNTN4–APLP2 crystal structures, we speculate that interacting CNTN and amyloid family members are able to form *cis* complexes. Consistent with this hypothesis, CNTN5 and APLP1 form coreceptor complexes at presynaptic sites in cultured hippocampal neurons (24). In the case of CNTNs, the formation of such complexes is important because their attachment to the cell surface by a GPI anchor precludes direct transmission of signals inside the cell. For example, CNTN5 forms a coreceptor complex with the type I transmembrane protein CNTNAP4 during the arborization of dendrites in a subpopulation of retinal ganglion cells (5) as well as the formation of inhibitory microcircuits in the mouse spinal cord (41). We thus believe that our structural data indicate how CNTNs bind to APPs when they co-opt their cytoplasmic regions in order to relay information across the plasma membrane using amyloid-dependent signal transduction pathways (8).

Among the questions remaining to be answered, the identity of the protein or proteins expressed on the surface of the nucleus of the optic tract that would help attract CNTN4–APP-expressing axons is perhaps the most intriguing. Ig superfamily proteins can mediate both homophilic and heterophilic interactions to mediate axon-target specificity, but the results of our protein-binding assays indicate that only CNTN2 binds homophilically (Fig. 2). In particular, CNTN5 is not involved in homophilic interactions (Fig. 2) but can mediate homophilic cell aggregation when expressed with its coreceptor CNTNAP4 (5). Thus, it is possible that a CNTN4–APP complex expressed on the surface of axons could interact with another CNTN4–APP complex expressed on the surface of target cells (Fig. 9*A*). Consistent with this hypothesis, APP has been found on the surfaces of both axons and dendrites in primary mouse cortical neurons (42). The existence of additional CNTN–amyloid pairs also raises the possibility that interactions can occur between the distinct combinations of CNTN3–5 with APP, APLP1, and APLP2. Another possible consideration is that APP is ubiquitously expressed throughout the nervous system in general and in retinal ganglion cells in particular (7), whereas the expression of CNTN4 is more restricted (4). As such, CNTN4 might play a more determining role than APP in guiding the topography of innervation for retinal ganglion cell axons so that a CNTN4-binding partner expressed specifically in the nucleus of optic tract could promote contacts with CNTN4-expressing axons (Fig. 9*B*). The receptor protein tyrosine phosphatase PTPRG, which associates with CNTN4 *via* its second and third Ig domains (31), could participate in these adhesive interactions. Overall, the work performed here provides tools to start deconstructing the targeting mechanism that CNTN4 and APP mediate together.

During the gene expression analyses we performed in zebrafish embryos, we detected the presence of *cntn4*, *aplp1*, and *aplp2* mRNAs in the olfactory placode and subsequently in olfactory pits where olfactory sensory neurons reside (43). These combined expression patterns, taken together with (i) the role that CNTN4–APP plays in targeting axon subpopulations to the mouse visual system (4) and (ii) the inappropriate innervation of glomeruli in the olfactory bulbs of mice lacking CNTN4 (3), lead us to speculate that CNTN4–APLP1 and/or CNTN4–APLP2 might be involved in the patterning of olfactory tissues. In line with this hypothesis, APLP2 is abundantly expressed in the axon terminals of olfactory sensory neurons where CNTN4 is also found (21, 44). Although altered olfactory circuitry was not reported in mice lacking either *Aplp1* or *Aplp2*, it is possible that such defects were not examined in detail in these animals (45) or that *Aplp1* and *Aplp2* function redundantly in the olfactory system. However, phenotypic analyses of mice lacking both *Aplp1* and *Aplp2* were not possible as these animals die soon after birth (45). Nevertheless, olfactory sensory neurons target glomeruli erroneously in mice overexpressing human APP bearing the Swedish mutations (46). This mutation renders human APP more susceptible to processing by β-secretase and thus increases the shedding of its extracellular region from the cell surface along with increased production of the Aβ fragment. In these experiments, sensory neurons expressing the odorant receptor MOR28 innervated multiple glomeruli, a defect that was observed in *Cntn4*^*−/−*^ mice (3). To be sure, the innervation defects in mice expressing the Swedish form of APP were attributed in part to the increased presence of the Aβ fragment (46). However, we speculate that the soluble form of the APP ectodomain may also interfere with the formation of complexes between CNTN4, APLPL1, or APLP2 at the surface of sensory neurons, thus leading to targeting defects. We believe that a reexamination of the connectivity in the olfactory tissues of mice lacking members of the amyloid family is warranted and may provide novel insights into the roles that CNTN4–amyloid complexes may play in establishing axon-target specificity.

## Experimental procedures

### Cloning

Complementary DNA (cDNA) fragments encoding the wildtype and mutated regions of FN1–FN3 regions from CNTNs along with BamHI and EcoRI sites at the 5′ and 3′ end, respectively, were purchased from Integrated DNA Technologies (IDT) or Genscript, digested with BamHI and EcoRI and ligated into the plasmid pT7HMP, a derivative of the pT7HMT plasmid that includes an human rhinovirus 3C protease site instead of a tobacco etch virus protease site (47). Likewise, cDNA fragments encoding the E1 domains of vertebrate amyloids were purchased from IDT and cloned into a derivative of the pET32 plasmid (31). The single-chain fusion protein of chicken CNTN3(FN1–FN2) and APP(E1) was created by adding the linker GGGSGGGS at the C terminus of E699 of chicken CNTN3 and fusing it to E19 of chicken APP. The single-chain fusion protein of zebrafish CNTN4(FN1–FN2) and APLP2(E1) was created by adding the linker GGGSGGGS at the C terminus of E802 of zebrafish CNTN4 and fusing it to I23 of zebrafish APLP2. Corresponding cDNA fragments were purchased from IDT and cloned into the pET32 derivative described previously. All purchased cDNA fragments were optimized for expression in bacteria. cDNA constructs encoding the complete extracellular regions of APP, APLP1, APLP2, CNTN2, CNTN6, GABBRI, and PTPRG were synthesized by Genscript and cloned into derivatives of the pLex2 vector to express these proteins as fusion proteins with human IgG1 Fc and chicken IgY Fc fused to a Twin-Strep tag (48). Fusion proteins of CNTN1 and CNTN3–5 with human IgG1 Fc have been described previously (34) and were transferred in the vector expressing the FcYTS tag. All plasmid constructs were verified by DNA sequencing.

### Protein expression and purification

Wildtype and mutated FN1–FN3 fragments of CNTN3–5 from chicken, human, mouse, and zebrafish were expressed as hexahistidine fusion proteins in *Escherichia coli* strain BL21(DE3) as described previously (34). The E1 domains of chicken, human, mouse, APP, zebrafish APPb, human APLP1, and zebrafish APLP2, as well as the fusion proteins CNTN3–APP and CNTN4–APLP2, were expressed with thioredoxin, a hexahistidine tag, and a human rhinovirus 3C protease site in *E. coli* strain Origami2(DE3) adapted from a procedure described by Dahms *et al.* (36). All proteins were purified *via* immobilized-metal affinity chromatography followed by cleavage with human rhinovirus 3C protease. Subsequent purification steps involved ion exchange and gel filtration chromatography. An additional step of heparin-affinity chromatography was added to this workflow to purify most of the E1 domains of APP, APLP1, APLP2 and their fusion proteins with CNTN3 or CNTN4. In our hands, human APP(E1) did not bind to a heparin-Sepharose column.

For biotinylation, proteins freshly purified from size-exclusion chromatography in gel filtration buffer (150 mM NaCl, 20 mM Na–Hepes, pH 7.5) were incubated with a 1:1 M ratio of EZ-Link NHS-PEG4-Biotin (Thermo Fisher Scientific) for 2 h on ice according to the manufacturer's instructions. To avoid problems linked to aggregation following concentration in centrifugal devices, the most concentrated fraction from the size-exclusion purification was selected for biotinylation. The reaction was quenched by adding Tris–HCl (pH 8.0) to a final concentration of 100 mM, and the protein was extensively dialyzed at 4 °C against 150 mM NaCl and 20 mM Na–Hepes (pH 7.5). The proteins were then stored at 4 °C until use.

### BLI experiments

Interactions between CNTN and amyloid family members were quantified at room temperature in 150 mM NaCl, 10 mM Na–Hepes (pH 7.5), 1 mg/ml bovine serum albumin, 0.02% (v/v) Tween-20 using a BLItz or Octet K2 system (Sartorius). Biotinylated E1 domains of APP, APLP1, and APLP2 (250 nM) were immobilized onto streptavidin tips (Sartorius). These tips were then incubated with purified CNTNs at a series of concentrations for 120 to 180 s during the association phase, by which time the signal had reached a plateau. The tips were then incubated in buffer only during the dissociation phase for 120 to 180 s. The signal was corrected by subtracting the background measured for the buffer only. In these experiments reported here, the observed dissociation rate constants attained or exceeded the accuracy limits of the instruments (*k*_off_ > 0.01 s^−1^). Thus, the dissociation constants (*K*_*D*_) were calculated by plotting the values of the maximal binding signal obtained at equilibrium against the concentration of CNTNs and fit using Prism 8 (GraphPad Software, Inc) to the equation: signal = max ∗ C/(*K*_*D*_ + C) (where max is the maximal CNTN binding and C is the concentration of CNTN). The results are reported as the average of at least three biological CNTN replicates.

The FN1–FN3 domains of zebrafish CNTN4 precipitated at concentrations above 40 μM, which hampered the binding of increasing concentrations of CNTN4 to immobilized APPb or APLP2. Thus, the FN1–FN3 domains of CNTN4 were biotinylated, immobilized on streptavidin sensors, and the binding of increasing concentrations of the E1 domains of zebrafish APPb and APLP2 to these tips was measured on an Octet K2 system (Sartorius). The dissociation constants, *K*_*D*_, were calculated as described previously, and results are reported as the average of at least three biological replicates APPb or APLP2.

### Crystallization and structure determination

All crystals were grown at 20 °C by hanging-drop vapor-diffusion method except for the complex of zebrafish CNTN4(FN1–FN3) and APPb(E1). Detailed crystallization and cryoprotection conditions can be found in Table S2. In the case of zebrafish CNTN4–APPb, complex crystals adventitiously grew within 2 weeks in the protein stock stored at 4 °C in 75 mM NaCl and 10 mM Na–Hepes (pH 7.5). Crystals were transferred to a solution of 30% (w/v) of PEG 3350, 150 mM NaCl, 20 mM Na–Hepes (pH 7.5) prior to freezing in liquid nitrogen. X-ray diffraction data were collected on beamlines 22-ID and 22-BM of the Advanced Photon Source at the Argonne National Laboratory. Diffraction data were processed using either HKL2000 or iMosflm/Aimless as implemented in the CCP4 package (49, 50, 51, 52). Ramachandran and geometry statistics for all models were validated using the Research Collaboratory for Structural Bioinformatics PDB validation server. Structures were determined by molecular replacement in PHASER as implemented by PHENIX (53) using the previously published crystal structures of the E1 domain of mouse APP and the FN1–FN3 domains of mouse CNTN3, mouse CNTN4, and human CNTN5 as search models (34, 36). The final models were obtained after several rounds of manual rebuilding in COOT (54) and refinement in PHENIX as well as refinement using PDB Redo (55). These models were validated using the Research Collaboratory for Structural Bioinformatics PDB validation server. Shape complementarity coefficients were calculated using *S*_C_ (39) as implemented by CCP4, whereas list of interacting residues and interface areas were obtained using the PISA server (56). Structural representations were generated using ChimeraX (57).

### AlphaScreen binding assays

The interactions between APP and CNTN family members were analyzed using an extracellular binding assay. Candidate proteins were fused with either human IgG1 Fc or Cν3–Cν4 domains of chicken IgY tagged with a Twin-Strep peptide (abbreviated FcYTS, (29)) and expressed in human embryonic kidney 293 cells. Conditioned media were dialyzed extensively against 150 mM NaCl and 20 mM Na–Hepes (pH 7.5) to remove traces of biotin prior to the experiments. At this stage, proteins can be used immediately or placed at −80 °C for long-term storage. To run the assay, aliquots (7.5 μl) of candidate proteins fused to Fc were pipetted into a 96-well plate followed by addition of an equal volume of candidate proteins fused to FcYTS. A solution of Strep-Tactin Alpha donor beads (37.5 μg/ml; PerkinElmer Life Sciences) and AlphaScreen Protein A acceptor beads (37.5 μg/ml; PerkinElmer Life Sciences) was prepared in 150 mM NaCl, 20 mM Na–Hepes (pH 7.5), 2 mM CaCl_2_, 2 mM MgCl_2_, 2 mg/ml bovine serum albumin, 0.2% (v/v) Triton X-100, and 0.2% (v/v) Tween-20. Aliquots (15 μl) of this solution were immediately added to the wells containing the candidate proteins. The well contents were then transferred to a 96-well half-area opaque microplates. After a 1-h incubation at room temperature, plates were analyzed on an EnSpire multimode plate reader (PerkinElmer Life Sciences). For each candidate protein, the intensities were normalized by dividing the measured luminescence signal by the signal obtained for the negative control that includes the candidate protein fused to the FcYTS tag and an Fc-only sample (Fig. S1). Experiments were then repeated with the tag swapped between the two candidate proteins (*i.e.*, APP-Fc *versus* CNTN4-FcYTS and APP-FcYTS *versus* CNTN4-Fc). We defined an interaction pair as one for which we measure a signal over background of at least two for each orientation (protein 1-Fc/protein 2-FcYTS and protein 2-Fc/protein 1-FcYTS). Protein pairs for which luminescence signals were identified in only one orientation were ignored.

### *In situ* hybridizations

Adult ∗AB zebrafish were raised under standard conditions and embryos were collected from natural crosses and staged according to standard protocols at 28.5 °C (58). All zebrafish experiments were conducted according to approved protocols (number: 1707-02) under the Institutional Animal Care and Use Committee at the University of Missouri—Kansas City. Antisense RNA *in situ* hybridizations were performed using the protocols standard in the field (59). We purchased full-length clones of *aplp2*, *cntn4*, and *cntn5* (Horizon Discovery) and used PCR to amplify cDNA fragments prior to synthesizing the probes. Fragments from *cntn4* (forward primer: 5′-TCTCTGTGGGCCTCCTCCGC-3′; reverse primer: 5′-TGCGCCAGGAGATCGAGCCT-3′ (60)) and *cntn5* (forward primer: 5′-CTGTCATGGAGCCACGGTGT-3′; reverse primer: 5′-TGAATCCACATCAAGTTGCC-3′) were cloned into the pCR4-TOPO using the TOPO TA cloning kit (Thermo Fisher Scientific) according to the manufacturer's instructions. We generated antisense riboprobes from PCR fragments (1476730) for *aplp1* (forward primer: 5′-ACCGCCACCACGGTCAAATAC-3′; reverse primer: 5′-CCAAGCTTCTAATACGACTCACTATAGGGAGACCCACGGTTAAACGTCTCCA-3′), *aplp2* (forward primer: 5′-CCCTGTGGCATCGATAAGTT-3′, reverse primer: 5′-CCAAGCTTCTAATACGACTCACTATAGGGAGACCGTACTGCCTCTTCCTCAG-3′ (19)), and *appb* (forward primer: 5′-CAGCCTCGGCCTCGGCAGGTG-3; reverse primer: 5′-CCAAGCTTCTAATACGACTCACTATAGGGAGAGTTCTGCATTTGCTCAAAGAAC-3′). The cDNA templates for *aplp1* and *appb* were synthesized by IDT. Labeling was detected using antifluorescein or antidigoxygenenin antibodies congregated to alkaline phosphatase F(ab)’_2_ fragments (Roche). Staining was developed using nitro blue tetrazolium and 5-bromo 4-chloro 3-indolyl phosphate substrates. Images were collected using a Zeiss Axio Imager.V6 and a 20× differential interference contrast objective. Images were processed using Affinity Photo (Serif) to adjust brightness and contrast using identical settings for all images.

## Data availability

The atomic coordinates and structure factors (PDB codes: 7MQY, 7MRK, 7MRM, 7MRN, 7MRO, 7MRQ, and 7MRS) have been deposited in the PDB (http://wwpdb.org/). All other data are contained within the article and supporting information.

## Supporting information

This article contains supporting information.

## Conflict of interest

The authors declare that they have no conflicts of interest with the contents of this article.
