# Supplementary material for: Members of the vertebrate contactin and amyloid precursor protein families interact through a conserved interface
Source: J Biol Chem. 2021 Dec 25;298(2):101541. doi: 10.1016/j.jbc.2021.101541 (PMC8808184; doi:10.1016/j.jbc.2021.101541)
Supplement: Supplemental Table S1 [file mmc1.docx]

**Supplementary Table 1 – Summary of Bio-Layer interferometry assays**

| **Amyloid**  **(E1)** | **CNTN**  **(FN1-FN3)** | **Average Kinetic K_D_ (μM)** | **Average K_on_**  **(M^-1^s^-1^)** | **Average K_off_**  **(s^-1^)** | **Average Steady State K_D_ (µM)** | **Biological**  **replicates** | **Instrument/Notes** |
| --- | --- | --- | --- | --- | --- | --- | --- |
| Chicken APP | Chicken CNTN3 | 182 ± 177 | 7250 ± 9900 | 0.90 ± 0.14 | 46.8 ± 34.7 μM | 3 | BLItz |
| Chicken APP | Chicken CNTN4 | 8.2 ± 6.3 | 33900 ± 25854 | 0.19 ± 0.05 | 5.4 ± 1.7 μM | 3 | BLItz |
| Chicken APP | Chicken CNTN4 T751A, V752A | 441 ± 370 | 10300 ± 15032 | 0.38 ± 0.45 | 20.9 ± 5.0 μM | 3 | BLItz |
| Chicken APP | Chicken CNTN4 Y781A, E786A | 330 ± 5206.3 | 5446 ± 4509 | 0.59 ± 0.44 | 115.3 ± 12.0 μM | 3 | BLItz |
| Chicken APP | Chicken CNTN4 T751A, V752A, Y781A, E786A | No binding | No binding | No binding | No binding | 4 | BLItz |
| Chicken APLP2 | Chicken CNTN4 | 26.6 ± 24.3 | 21600 ± 15139 | 0.33 ± 0.08 | 28.2 ± 7.2 | 3 | BLItz |
| Chicken APLP2 | Chicken CNTN4 T751A, V752A, Y781A, E786A | No binding | No binding | No binding | No binding | 2 | BLItz |
| Human APP | Human CNTN3 | 403 ± 284 | 2561 ± 1262 | 0.79 ± 0.07 | 63.3 ± 14.3 μM | 4 | BLItz |
| Human APP | Human CNTN4 | 18.7 ± 6.1 | 19200 ± 7192 | 0.33 ± 0.04 | 8.0 ± 0.9 μM | 3 | BLItz |
| Human APP | Human CNTN5 | No binding | No binding | No binding | No binding | 3 | BLItz. Lack of binding possibly due to aggregation |
| Human APLP1 | Human CNTN3 | 4.5 ± 0.2 | 2190 ± 1722 | 0.10 ± 0.01 | 9.1 ± 0.3 μM | 3 | BLItz |
| Human APLP1 | Human CNTN4 | 0.4 ± 0.08 | 64500 ± 11363 | 0.03 ± 0.001 | 1.5 ± 0.3 μM | 3 | BLItz |
| Human APLP1 | Human CNTN5 | 9.8 ± 1.5 | 17700 ± 3304 | 0.17 ± 0.48 | 10.9 ± 1.3 μM | 3 | BLItz |
| Human APLP2 | Human CNTN4 | 4.8 ± 1.02 | 43000 ± 13807 | 0.2 ± 0.03 | 6.3 ± 1.8 μM | 3 | BLItz |
| Human APLP2 | Human CNTN5 | 36.6 ± 23.3 μM | 9440 ± 2501 | 0.37 ± 0.301 | 38.6 ± 14.6 μM | 3 | BLItz |
| Zebrafish APPb | Zebrafish CNTN4 | 0.11 ± 0.05 | 170000 ± 103773 | 0.03 ± 0.01 | 401.1 ± 10.0 nM | 3 | Octet K2 |
| Zebrafish APPb | Zebrafish CNTN5 | 64.4 ± 23.2 | 9561 ± 1935 | 0.59 ± 0.11 | 26.8 ± 13.6 μM | 3 | BLItz |
| Zebrafish APLP2 | Zebrafish CNTN4 | 100 ± 95.2 | 79000 ± 136799 | 0.16 ± 0.26 | 21.4 ± 12.3 μM | 3 | Octet K2 |
| Mouse APP | Mouse CNTN3 | 2.9 ± 1.1 | 56200 ± 1175 | 0.15 ± 0.04 | 6.3 ± 2.9 μM | 4 | BLItz |
| Mouse APP | Mouse CNTN4 | 0.2 ± 0.03 | 110000 ± 16975 | 0.02 ± 0.01 | 0.22 ± 0.06 μM | 4 | BLItz |
| Mouse APP | Mouse CNTN5 | 10.8 ± 2.1 | 17500 ± 5236 | 0.18 ± 0.03 | 16.2 ± 11.8 μM | 4 | BLItz |
| Mouse APP | Mouse CNTN4 | 0.25 ± 0.00 | 175600 ± 1058 | 0.04 ± 0.00 | 0.28 ± 0.02 | 3 | Octet K2 |
| Mouse APP | Mouse CNTN4 M748I | 1.5 ± 0.2 | 145967 ± 9304 | 0.20 ± 0.02 | 1.6 ± 0.02 | 3 | Octet K2 |
| Mouse APP | Mouse CNTN4 M748K, Y760F, F762Y | 1.1 ± 0.1 | 167600 ± 5756 | 0.20 ± 0.01 | 1.5 ± 0.02 | 3 | Octet K2 |
| Mouse APP | Mouse CNTN4 T750K, V751M | 276.3 ± 22.8 | 4277 ± 354 | 1.2 ± 0.01 | 65.6 ± 5.1 | 3 | Octet K2 |
| Mouse APP | Mouse CNTN4 M748K, T750K, V751M Y760F, F762Y, M748 | 185.3 ± 3.2 | 6936 ± 15 | 1.3 ± 0.02 | 54.0 ± 2.3 | 3 | Octet K2 |
| Mouse APP A126V | Mouse CNTN4 | 1.3 ± 0.04 | 159867 ± 4829 | 0.20 ± 0.00 | 1.1 ± 0.04 | 3 | Octet K2 |
| Mouse APP A126V | Mouse CNTN4 M748I | 120.6 ± 37.2 | 11722 ± 3028 | 1.3 ± 0.02 | 34.2 ± 0.7 | 3 | Octet K2 |
| Mouse APP A126V | Mouse CNTN4 M748K, Y760F, F762Y | 1.9 ± 0.1 | 162867 ± 10108 | 0.30 ± 0.01 | 2.0 ± 0.1 | 3 | Octet K2 |
| Mouse APP A126V | Mouse CNTN4 T750K, V751M | 178.3 ± 228.5 | 1178 ± 1972 | 0.5 ± 0.9 | 73.1 ± 1.3 | 3 | Octet K2 |
| Mouse APP A126V | Mouse CNTN4 M748K, T750K, V751M Y760F, F762Y, M748 | 167.1 ± 83.7 | 5011 ± 4314 | 1.1 ± 0.9 | 69.7 ± 4.4 | 3 | Octet K2 |
