# Supplementary material for: Members of the vertebrate contactin and amyloid precursor protein families interact through a conserved interface
Source: J Biol Chem. 2021 Dec 25;298(2):101541. doi: 10.1016/j.jbc.2021.101541 (PMC8808184; doi:10.1016/j.jbc.2021.101541)
Supplement: Supplemental Table S2 [file mmc2.docx]

**Supplementary Table 2 – Crystallization and cryo-preservation conditions**

| Protein | Buffer | Crystallization Condition | Cryoprotectant |
| --- | --- | --- | --- |
| Chicken CNTN4•APP (50 µM) | 50 mM NaCl,  5 mM Na-HEPES pH 7.5 | 150 mM (NH_4_)_2_SO_4_, 50 mM Na-cacodylate pH 6.5, 30% (v/v) PEG 550 MME | Frozen in mother liquor |
| Chicken CNTN3-APP fusion (346 µM) | 50 mM NaCl,  10 mM Na-HEPES pH 7.5 | 100 mM Na-acetate pH 5.5, 1.1 M Li_2_SO_4_, 3% (w/v) sucrose | 100 mM N Na-acetate pH 5.5, 1.1M Li_2_SO4, 20 % (v/v) ethylene glycol, 10% (v/v) PEG 400 |
| Zebrafish CNTN4•APPb (120 µM) | 75 mM NaCl,  10 mM Na-HEPES pH 7.5 | 75 mM NaCl,  10 mM Na-HEPES pH 7.5 | 150 mM NaCl, 20 mM HEPES pH 7.5, 30% (w/v) PEG 3,350 |
| Zebrafish CNTN4-APLP2 fusion (213 µM) | 75 mM NaCl,  10 mM Na-HEPES pH 7.5 | 100 mM Na-acetate pH 5.2,  30% (w/v) PEG 8,000 | Frozen in mother liquor |
| Chicken CNTN4 (T751A, V752A, Y781A, E786A) (113 µM) | 30 mM NaCl | 4.2M NaCl, 2 (v/v) % Glycerol | Frozen in mother liquor |
| Zebrafish CNTN4  (213 µM) | 75mM NaCl, 10mM Na-HEPES pH 7.5 | 100mM Na-acetate pH 5.5,  30% (w/v) PEG 8,000 | 25mM Na-acetate pH 5.2,  25mM Na-acetate pH 5.5,  30% PEG 8,000 |
| Mouse APP•CNTN5  (124 µM) | 75mM NaCl, 10mM Na-HEPES pH 7.5 | 100mM Na-citrate pH 5.5, 10% Isopropanol, 25% (w/v) PEG 400 | Frozen in mother liquor |
